# Four New Picrotoxane-Type Sesquiterpenes From *Dendrobium nobile* Lindl

**DOI:** 10.3389/fchem.2019.00812

**Published:** 2019-11-29

**Authors:** Pei Wang, Xin Chen, Hao Wang, Shengzhuo Huang, Caihong Cai, Jingzhe Yuan, Guoliang Zhu, Xinglian Xu, Wenli Mei, Haofu Dai

**Affiliations:** ^1^Hainan Key Laboratory of Research and Development of Natural Product From Li Folk Medicine, Institute of Tropical Bioscience and Biotechnology, Chinese Academy of Tropical Agricultural Sciences, Haikou, China; ^2^Nanjing Agricultural University, Nanjing, China; ^3^East China University of Science and Technology, Shanghai, China

**Keywords:** *Dendrobium nobile* Lindl, picrotoxane, sesquiterpenes, α-glycosidase inhibitor, cytotoxicity

## Abstract

Four picrotoxane-type sesquiterpenes, dendroterpene A–D (**1**–**4**), together with four known compounds (**5**–**8**), were isolated from the stems of *Dendrobium nobile* Lindl. Their structures were elucidated by spectroscopic analysis, X-ray diffraction analysis, analysis of the ECD data according to the Klyne's lactone sector rule, and quantum ECD calculation. Compounds **1** and **2** are two new picrotoxane-type sesquiterpenes with a new carbon skeleton containing a formamide group, which may be derived from the previously reported dendrobiumane B skeleton by the C(9)-C(11) carbon bond cleavage. Compounds **3**, **5**, **6**, and **8** exhibited inhibitory activity against α-glycosidase. Compounds **5** and **6** were cytotoxic against SGC-7901, K562, A549, BEL-7402, and Hela cell lines.

## Introduction

*Dendrobium nobile* Lindl, the plant belongs Orchidaceae family, is one of three major plant sources of the traditional Chinese medicine “Shi Hu” in Chinese (edition 2005), which are used as a tonic to nourish the stomach and promote the production of body fluid recorded in the Chinese Pharmacopeia (Jiangsu New Medical College, 1986). The previous reports showed a series of chemical constituents isolated from *D. nobile* including sesquiterpenes (Zhang et al., 2007a), alkaloids (Liu and Zhao, 2003; Meng et al., 2017), bibenzyls derivatives (Zhang et al., 2007b), and glucosides (Zhao et al., 2001), some of which exhibited antitumor (Zhou et al., 2016), anti-inflammatory (Hwang et al., 2010), and immunomodulatory activities (Zhao et al., 2001).

Picrotoxane-type sesquiterpenes were one of main constituents of *D. nobile*, which exhibited the angiogenesis effect against sunitinibinduced damage (Meng et al., 2017) and inhibitory activity of nerve growth factor mediated neurite outgrowth (Leon et al., 2019). Our phytochemical investigation on the EtOAc extract of the stems of *D. nobile* led to the isolation of four new picrotoxane-type sesquiterpenes, dendroterpene A–D (**1**–**4**), along with four known compounds, nobilin E (**5**) (Zhang et al., 2007b), dendrocandin V (**6**) (Xiao et al., 2017), *S*(+)-dehydrovomifoliol (**7**) (Kato et al., 1977) and di-[2-(4-hydroxyphenyl)] ethyl ether (**8**) (Krysin et al., 2010). Compounds **1** and **2** are two new picrotoxane-type sesquiterpenes with a new carbon skeleton containing a formamide group, which may be derived from the previously reported dendrobiumane B skeleton by the C(9)-C(11) carbon bond cleavage. In this report, the isolation, structure elucidation and bioactivities of these compounds are described.

## Materials and Methods

### General Experimental Procedures

Optical rotations were measured on a MCP 5100 modular compact polarimeter (Anton Paar, America). IR spectra were taken on a Nicolet 380 FT-IR instrument (Thermo, USA) as KBr discs. NMR spectra were recorded on a Bruker Avance III 500 MHz NMR spectrometer (Bruker, German). ESIMS and HRESIMS were recorded with amaZon SL (Bruker) or Compact QqTOF (Bruker). ECD spectra were measured by APL Chiascan (Applied Photophysics Ltd., England). Semi-preparative HPLC was carried out using a C_18_ column (Cosmosil-pack, 10 × 250 mm, 5 μm, 4 mL/min, Nacalai Tesque). C_18_ gel (20–45 μm, Fuji SilysiSa Chemical Co., Ltd., Greenville, NC, UA), Silica gel (60–80, 200–300 mesh, Qingdao Marine Chemical Co., Ltd., Qingdao, China), Sephadex LH-20 (Merck, Kenilworth, NJ, USA) were used for column chromatograghy. α-glycosidase (Sigma-Aldrich, UA) used for the bioactivity was derived from yeast and its EINECS (EC) unmber is 3.2.1.20.

### Plant Material and Extraction

The fresh stems of *D. nobile* were collected from Shishan town in May 2018, Hainan, China. After drying, the dried stems (13.0 kg) were shattered. Then, the samples were extracted by 95% ethyl alcohol three times and for 5 days each time. The EtOH extract (716.2 g) was dissolved in H_2_O and extracted three times by petroleum ether (8.0 L), EtOAc (8.0 L), and n-butyl alcohol (8.0 L) successively.

### Isolation

The EtOAc extract (87.9 g) was separated into 16 fractions (Fr.1–Fr.16) by a silica gel column (200–300 mesh) using a gradient of petroleum ether-EtOAc (v/v, 20:1– 0:1) and then acetone. Fr.9 (10.0 g) was further separated into 20 fractions (Fr.9-1–Fr.9-20) by ODS column. Fr.9-1 (30.0 mg) was purified by semipreparative HPLC (20% MeOH/H_2_O) to yield **8 (**1.4 mg; *t*_R_ 12.1 min). Fr.9-6 (112.0 mg) was purified by semipreparative TLC eluting with petroleum ether-acetone (v/v 3:1) to obtain compound **7** (2.7 mg). Fr.9-7 (372.0 mg) was purified by a silica gel column eluting with petroleum ether-CHCl_3_-MeOH (v/v/v 200:50:1) to yield compound **3** (4.2 mg). Compound **4** (2.0 mg) was obtained by recrystallization from Fr.9-8 (580.3 mg). Fr.9-10 (103.0 mg) was further purified by a silica gel column eluting with petroleum ether-acetone (v/v 8:1) to give **1** (13.0 mg). Fr.10 (9.0 g) was separated by ODS column to get 50 fractions (Fr.10-1–Fr.10-50). Compound **2** (100.2 mg) was obtained by recrystallization from Fr.10-14 (326.0 mg). Fr.10-38 (184.0 mg) was purified by Sephadex LH-20 eluting with MeOH to give **5** (15.0 mg). Fr.11 (9.0 g) was separated into three fractions (Fr.11-1–Fr.11-3) by Sephadex LH-20 eluting with MeOH. Fr.11-3 (80.1 mg) was purified by ODS column eluting with 65% MeOH/H_2_O to yield compound **6** (20.9 mg).

### ECD Calculation

The calculations were performed by using the density functional theory (DFT) as carried out in the Gaussian 03 (Frisch et al., 2004). The preliminary conformational distributions search was performed by Sybyl-X 2.0 software. All ground-state geometries were optimized at the B3LYP/6-31G(d) level. Solvent effects of methanol solution were evaluated at the same DFT level by using the SCRF/PCM method (Cammi and Tomasi, 1995). TDDFT at B3LYP/6-31G(d) was employed to calculate the electronic excitation energies and rotational strengths in methanol (Gross et al., 1996).

### Characterization of Compounds 1–4

Dendroterpene A (**1**): Colorless crystals; [${\left[\alpha \right]}_{\text{D}}^{25}$] +149.9 (*c* 0. 1, MeOH); ECD (MeOH) λ_max_ (Δε) 223 (+2.28); IR (KBr) ν_max_ 3350, 2927, 2856, 1761, 1683, 1118, 1024 cm^−1^; m.p. 185–186 °C; ^1^H and ^13^C NMR (see Table 1); HRESIMS *m*/*z* 286.1417 [M + Na]^+^ (calcd. for C_15_H_21_NO_3_Na: 286.1414).

**Table 1:** ^1^H (500 MHz) and ^13^C (125 MHz) NMR Data of compounds **1**–**4**.

**No**.	**1^a^**		**2^b^**		**3^c^**		**4^c^**	
	**δ_**C**_**	**δ_**H,**_ mult. (*J* in Hz)**	**δ_**C**_**	**δ_**H,**_ mult. (*J* in Hz)**	**δ_**C**_**	**δ_**H,**_ mult. (*J* in Hz)**	**δ_**C**_**	**δ_**H,**_ mult. (*J* in Hz)**
1	52.5, C	–	53.3, C	–	47.8, C	–	50.4, C	–
2	51.2, CH	4.24, s	49.0, CH	4.18, d, (9.7)	81.5, CH	4.28,d, (3.6)	81.6, CH	4.25, d, (3.6)
3	84.5, CH	4.34, brd, (5.5)	83.2, CH	4.33, brd, (5.6)	77.9, CH	4.75,dd, (3.6, 5.6)	77.5, CH	4.79, dd, (3.6, 5.1)
4	52.5, CH	2.02, m	52.3, CH	2.01, ddd, (4.7, 4.7, 11.2)	50.9, CH	2.29,ddd, (5.0,5.0,11.7)	51.1, CH	2.27, m
5	46.1, CH	2.28, t, (4.3)	51.0, CH	2.47, d, (4.7)	43.4, CH	2.57, dd, (4.3,5.6)	43.2, CH	2.57, dd, (4.3, 5.8)
6	42.5, CH	2.33, m	80.6, C	–	43.6, CH	2.20, m	44.9, CH	2.31, m
6-OH	–	–	–	5.40, brs	–	–	–	–
7	36.9, CH_2_	2.69, ddd, (2.3, 2.3, 7.3,17.1) 2.35, m	46.1, CH_2_	2.64, brd, (15.1) 2.52, brd, (15.1)	32.4, CH_2_	2.15, m	29.7, CH_2_	2.07, m; 2.36, m
8	130.3, CH	5.51, dd, (2.3, 5.5)	137.3, CH	5.79, brd, (6.3)	27.8, CH_2_	2.01, m; 2.14, m	38.5, CH_2_	1.82, m; 2.28, m
9	136.6, CH	5.63, m	127.7, CH	5.47, d, (6.3)	55.2, CH	2.77,t, (9.4)	85.1, C	–
10	27.3, CH_3_	1.04, s	22.3, CH_3_	0.96, s	31.3, CH_3_	1.52, s	24.3, CH_3_	1.43, s
11	163.7, CH	8.09, s	161.7, CH	8.12, s	177.1, C	–	177.2, C	–
12-NH	–	–	–	8.51, d, (9.6)	–	–	–	–
13	26.4, CH	1.84, m	26.4, CH	2.36, m	24.8, CH	1.75, m	24.8, CH_3_	1.84, m
14	20.1, CH	0.95, d, (6.6)	22.2, CH_3_	1.00, d, (6.1)	20.6, CH_3_	1.04, d, (6.5)	20.7, CH_3_	1.04, d, (6.5)
15	21.0, CH_3_	0.93, d, (6.6)	20.3, CH_3_	0.89, d, (6.6)	21.4, CH_3_	1.02, d, (6.5)	21.5, CH_3_	1.02, d, (6.6)
16	180.3, C	–	176.5, C	–	177.5, C	–	177.5, C	–

a measured in CD_3_OD;

b measured in DMSO-d_6_;

c *measured in CDCl_3._*.

Dendroterpene B (**2**): Colorless crystals; [${\left[\alpha \right]}_{\text{D}}^{25}$] +10.0 (*c* 0.1, MeOH); ECD (MeOH) λ_max_ (Δε) 207 (+4.77); IR (KBr) ν_max_ 3414, 2964,2926, 2861, 1764, 1666, 1223, 1021 cm^−1^; m.p. 200–201°C; ^1^H and ^13^C NMR (see Table 1); HRESIMS *m/z* 302.1376 [M + Na]^+^ (calcd. for C_15_H_21_NO_4_Na: 302.1363).

Dendroterpene C (**3**): White powder; [${\left[\alpha \right]}_{\text{D}}^{25}$] +16.0 (*c* 0.1, MeOH); ECD (MeOH) λ_max_ (Δε) 225 (+ 1.16), IR (KBr) ν_max_ 3447, 2928, 1777, 11276, 1111, 1036 cm^−1^; ^1^H and ^13^C NMR (see Table 1); HRESIMS *m/z* 287.1281 [M + Na]^+^ (calcd. for C_15_H_20_O_4_Na:287.1254).

Dendroterpene D (**4**): Colorless crystals; [${\left[\alpha \right]}_{\text{D}}^{25}$] +139.9 (*c* 0.1, MeOH); IR (KBr) ν_max_ 3414, 2930, 1629, 1108, 1028 cm^−1^; m.p. 167–168 °C; ^1^H and ^13^C NMR (see Table 1); HRESIMS *m*/*z* 303.1228 [M + Na]^+^ (calcd. for C_15_H_20_O_5_ Na: 303.1203).

### X-Ray Crystallographic Data of 1, 2, and 4

Compound **1** was obtained as colorless crystals with molecular formula of C_15_H_21_NO_3_ from MeOH. Space group *P2*_1_*2*_1_*2*_1_, *a* = 7.7078(5) Å, *b* = 10.1533 (7) Å, *c* = 17.8721(12) Å, α = 90.00°, β = 90.00(10)°, γ = 90.00°, *V* = 1398.66 (16) Å^3^, *Z* = 4, *D*_calcd_ = 1.251 g/cm^3^, μ(CuKα) = 0.7000 mm^−1^, and F(000) = 568, *T* = 293(2) K, crystal size 0.32 × 0.20 × 0.12 mm, *R*_1_ = 0.0445(*I*>*2sigma(I), wR*_2_ = 0.1054 (all data), Flack parameter = 0.0(4). 3147 reflections measured, 2119 unique (*R*_int_ = 0.0213, *R*_sigma_ = N/A) which were used in all calculations. The structure was solved by direct methods (SHELXS-97) and expanded using Fourier techniques (SHELXL-97). Crystallographic data (excluding structure factors) for structure **1** in this paper have been deposited in the Cambridge Crystallographic Data Center as supplementary publication number CCDC 1943060.

Compound **2** was obtained as colorless crystals with molecular formula of C_15_H_21_NO_4_ from MeOH. Space group *P*2_1_, *a* = 7.7610(4) Å, *b* = 8.8111 (4) Å, *c* = 10.3388(6) Å, α = 90.00°, β = 104.463(5)°, γ = 90.00°, *V* = 684.59 (6) Å^3^, *Z* = 2, *D*_calcd_ = 1.300 g/cm^3^, μ(CuKα) = 0.771 mm^−1^, *F*(000) = 300, crystal size 0.34 × 0.27 × 0.13 mm, *R*_1_ = 0.0390(*I*>*2sigma(I), wR*_2_ = 0.1028 (all data), Flack parameter = 0.0(4). 3304 reflections measured, 2070 unique (*R*_int_ = 0.0259, *R*_sigma_ = N/A) which were used in all calculations. The structure was solved by direct methods (SHELXS-97) and expanded using Fourier techniques (SHELXL-97). Crystallographic data (excluding structure factors) for structure **2** in this paper have been deposited in the Cambridge Crystallographic Data Center as supplementary publication number CCDC 1943062.

Compound **4** was obtained as colorless crystals with molecular formula of C_15_H_21_NO_3_ from MeOH. Space group *P*2_1_, *a* = 7.5120(3) Å, *b* = 13.6611(7) Å, *c* = 13.9060(5) Å, α = 90.00°, β = 90.00(10)°, γ = 90.00°, *V* = 1427.06(11) Å^3^, *Z* = 4, *D*_calcd_ = 1.360 mg/m^3^, μ(CuKα) = 0.842 mm^−1^, *F*(000) = 600, crystal size 0.327 × 0.22 × 0.15 mm. *R*_1_ = 0.0368(*I*>*2sigma(I), wR*_2_ = 0.0938 (all data), Flack parameter = 0.1(3). 2227 reflections measured, 1628 unique (*R*_int_ = 0.0204, *R*_sigma_ = N/A) which were used in all calculations. The structure was solved by direct methods (SHELXS-97) and expanded using Fourier techniques (SHELXL-97). Crystallographic data (excluding structure factors) for structure **4** in this paper have been deposited in the Cambridge Crystallographic Data Center as supplementary publication number CCDC 1943065.

### Bioassay for α-Glycosidase Inhibitory Activity

The method optimized by Jong et al. (2007) was performed *in vitro* to test the α-glucosidase inhibitory activity of compounds **1**–**8**. Acarbose was used as positive control.

### Bioassay for Cytotoxicity

The MTT method optimized by Mosmann (1983) was performed *in vitro* to test the cytotoxic activity of compounds **1**–**8**. Cisplatin was used as a positive control.

## Results and Discussion

### Identification of Compounds 1-4

Compound **1** was obtained as colorless crystals with the molecular formula as C_15_H_21_NO_3_, which was determined based on the pseudo-molecular ion peak at *m/z* 286.1417 [M + Na]^+^ (calcd. for C_15_H_21_NO_3_Na: 286.1414) in the HRESIMS spectrum. Analysis of its 1D and HSQC NMR spectra (see Supplementary Figures 1, 2, and 4 in the Supplementary Material) revealed two olefinic methines (δ_C/H_ 136.6/5.63 and δ_C/H_ 130.3/5.51), three methyls (δ_C/H_ 27.3/1.04, 21.0/0.93 and 20.1/0.95), one methylene (δ_C/H_ 36.9/2.69, 2.35), six sp^3^ methines (δ_C/H_ 84.5/4.34, 52.5/2.02, 51.2/4.24, 46.1/2.28, 42.5/2.33, and 26.4/1.84) with one oxygenated, one quaternary carbon (δ_C_ 52.5) and two ester or amide carbonyl groups (δ_C_ 180.3 and δ_C_ 163.7). The above data and sequential COSY correlations (Figure 2 and Supplementary Figure 3 in the Supplementary Material) from H-2 to H-9, as well as from H-4 to H_3_-14 and H_3_-15 through H-13, along with the HMBC correlations (Figure 2 and Supplementary Figure 5 in the Supplementary Material) from H_3_-10 to C-2, C-6 and C-9, from H-2 to C-6 and C-9, from H-3 and H-6 to C-16, and from H-5 to C-1, revealed a picrotoxane-type sesquiterpene skeleton (Zhao et al., 2003). A detailed comparison of the above data with those of the previously reported dendrobiumane C (Zhao et al., 2003) disclosed high similarity. The differences between them were that the hydroxymethyl group CH_2_-11 (δ_C/H_ 59.8/4.04, 4.27) and the oxymethine group CH-2 (δ_C/H_ 73.1/3.67) in dendrobiumane C were replaced by a hydrogen atom H-9 (δ_H_ 5.51) and a methine CH-2 (δ_C/H_ 51.2/4.24) linked with a formamide group in **1**, respectively, as evidence by COSY correlation from H-9 to H-8, along with the HMBC correlations from H-2 to C-9 and C-11, from H-9 to C-6 and C-10, as well as from H_3_-10 to C-2 and C-6. The ROESY correlations (Figure 3 and Supplementary Figure 6 in the Supplementary Material) from H_3_-10 to H-2, H-6 and H-13, and from H-2 and H-6 to H-13 suggested that H-2, H-6 and H_3_-10 were on the same face of the six-member ring, while H-4 was on the face opposite to them. Due to the complex bridge-ring lactone skeleton, the chemical structure models analysis of **1** displayed the only possibility of the relative configurations of C-3 and C-5 is that H-3 and H-5 were on the same face of six-member ring with H-2, H-6, and H_3_-10 when the relative configurations of C-1, C-2, C-4 and C-6 were confirmed. To support the above structure elucidation and determine the absolute configuration of **1**, a single-crystal X-ray diffraction pattern was obtained using the anomalous scattering of Cu Kα radiation (Figure 4), allowing an explicit assignment of the absolute configuration of **1** as 1*S*, 2*S*, 3*R*, 4*S*, 5*R*, and 6*S*. In order to confirm the absolute configuration assignment, Lactone sector rule (Jexkings et al., 1965) based on ECD data was used. The molecule was viewed from the line on the plane of the ester group along the bisectrix of the O–C = O angle, i.e., as shown in Figure 5 for 4*S*. The functional group at C-4 lying in the back upper right sector was responsible for the positive CD Cotton effect resulted from n–π^*^ transition of lactone, which was well in accordance with the positive Cotton effect at λ_max_ 223 nm of **1** (Supplementary Figure 24 in Supplementary Material). Hence, compound **1**, named as dendroterpene A, was determined to be a picrotoxane-type sesquiterpene.

**Figure 1:**
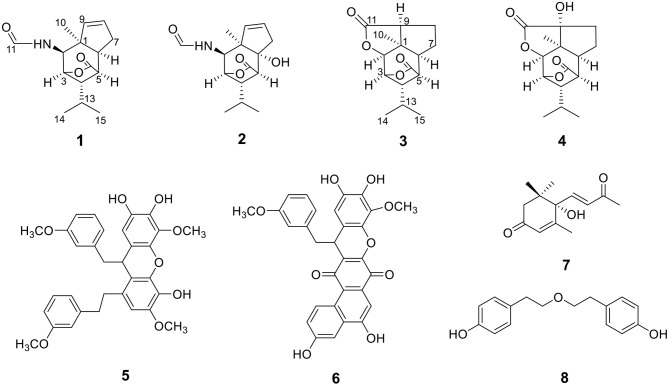
The structures of compounds **1–8**.

**Figure 2:**
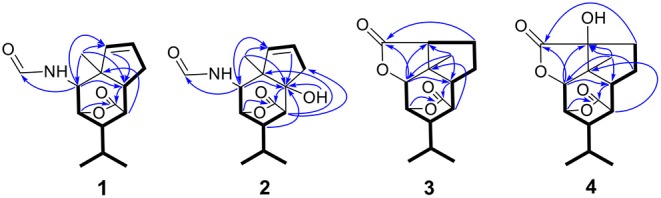
Key HMBC (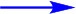) and ^1^H–^1^H COSY (−) correlations of compounds **1**–**4**.

**Figure 3:**
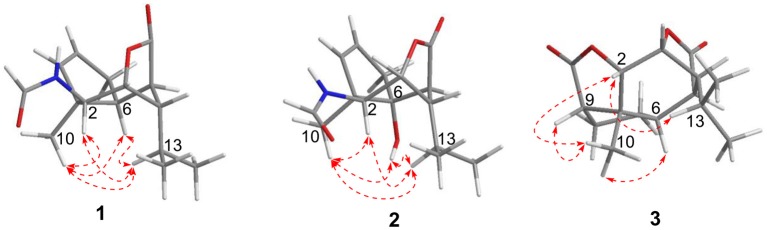
ROESY (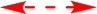) correlations of compounds **1**–**3**.

**Figure 4:**
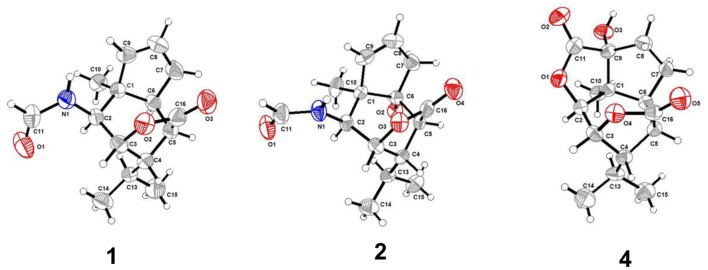
Molecular plots of compounds **1**, **2**, and **4**.

**Figure 5:**
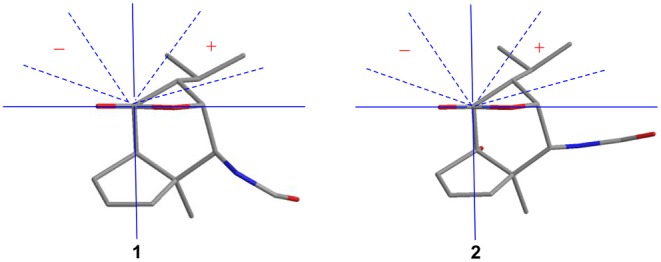
Application of lactone sector rule to compounds **1** and **2**.

Compound **2** was also isolated as colorless crystals. The HRESIMS (*m/z* 302.1376 [M + Na]^+^, calcd. for C_15_H_21_NO_4_Na: 302.1363) indicated a molecular formula of C_15_H_21_NO_4_. The 1D and 2D NMR data of **2** (Table 1 and Figure 2) were very similar to those of **1**, except that an oxygenated quaternary carbon C-6 (δ_C_ 80.6) in **2** replaced signals corresponding to CH-6 in **1** (δ_C/H_ 42.5/2.33). In the HMBC spectrum (Figure 2 and Supplementary Figure 11 in the Supplementary Material) of **2**, correlations from H-2, H-4, H-8, H-9, 6-OH, and H_3_-10 to the oxygenated quaternary carbon C-6, together with the molecular formula, suggested that the H-6 in **1** was oxygenated to a hydroxylated quaternary carbon in **2**. The H-2, 6-OH and H_3_-10 were on the same face of the six-member ring, and the H-4 was on the opposite face in **2**, as evidence by the ROESY correlations (Figure 3 and Supplementary Figure 12 in the Supplementary Material) of H_3_-10 with H-2, H-13 and 6-OH, and of H-2 and 6-OH with H-13. The relative configurations of C-3 and C-5 in **2** were identical with those of **1** according to the chemical structure models analysis of **2**.

The above assignment was further confirmed by a single-crystal X-ray diffraction pattern obtained using the anomalous scattering of Cu Kα radiation (Figure 4), which also led to an unambiguous assignment of the absolute configuration of **2** as 1*R*, 2*S*, 3*R*, 4*S*, 5*S*, and 6*R*. The absolute configuration of **2** was also confirmed by Lactone sector rule based on ECD data (Jexkings et al., 1965) (Figure 5 and Supplementary Figure 24 in Supplementary Material). Therefore, compound **2** was also identified as a picrotoxane-type sesquiterpene, and named as dendroterpene B.

**Figure 6:**
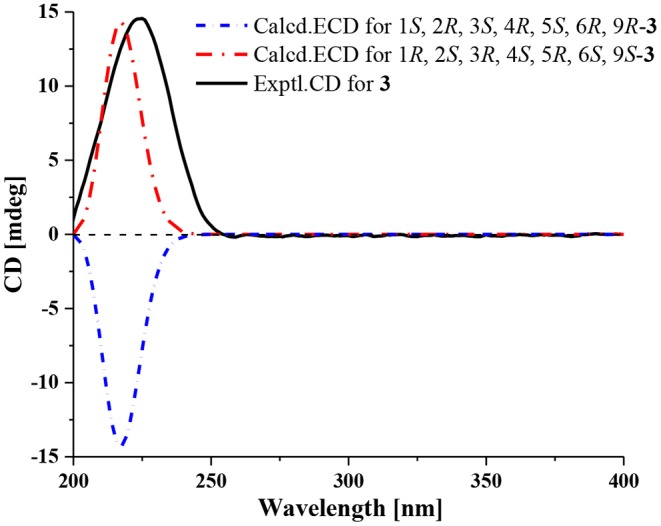
Measured ECD and TD-DFT calculated ECD curves of compound **3**.

Compound **3** has the molecular formula of C_15_H_20_O_4_ based on HRESIMS (*m/z* 287.1281 [M + Na]^+^, calcd for C_15_H_20_O_4_Na:287.1254), suggesting six degrees of unsaturation. 1D NMR and HSQC data displayed three methyls (δ_C/H_ 31.3/1.52, 21.4/1.02 and 20.6/1.04), two methylenes, seven methines including two oxygenated ones (δ_C/H_ 81.5/4.28 and 77.9/4.75), three quaternary carbons including two ester carbonyl carbons (δ_C_ 177.5 and 177.1). The above data of **3** were very similar to those of the previously reported dedrobiumane E (Zhao et al., 2003). Their structural differences were that two oxygenated quaternary carbons C-6 (δ_C_ 76.8) and C-9 (δ_C_ 83.8) and two oxygenated methine groups CH-7 (δ_C/H_ 65.2/4.05) and CH-8 (δ_C/H_ 60.1/3.86) in dedrobiumane E were replaced by two sp^3^ methines (δ_C/H_ 43.6/2.20 and δ_C/H_ 55.2/2.77) and two sp^3^ methylenes (δ_C/H_ 32.4/2.15 and δ_C/H_ 27.8/2.01, 2.14) in **3**, as proved by the sequential COSY correlations of H-5/H-6/H-7/H-8/H-9, along with the detail interpretation of the HMBC correlations from H-2 to C-6, C-9, and C-11, from H_3_-10 to C-2, C-6, and C-9, from H-3 and H-6 to C-16, from H-5 to C-1, as well as from H-8 to C-11 (Figure 2). The ROESY experiments (Figure 3 and Supplementary Figure 18 in the Supplementary Material) displayed the correlations from H_3_-10 to H-2, H-6, and H-9, as well as the correlations between H-2 and H-13, indicating H-2, H-6, H-9, and H_3_-10 were on the same face, while H-4 was on the face opposite to them. The relative configurations of C-3 and C-5 in **3** were also determined to be consistent with those of **1** and **2** through the same method. The calculated ECD curve for **3** matched well with the experimental one (Figure 6), assigning the absolute configurations of **3** as 1*R*, 2*S*, 3*R*, 4*S*, 5*R*, 6*S*, and 9*S* (Wang et al., 2014). Thus, the structure of **3** was established as picrotoxane-type sesquiterpene shown in Figure 1, and named as dendroterpene C.

Compound **4**, colorless crystals, the molecular formula of which was established to be C_15_H_20_O_5_ according to the pseudo-molecular ion peak at *m/z* 303.1228 [M + Na]^+^ (calcd. for C_15_H_20_O_5_Na: 303.1203) in the HRESIMS spectrum. The similarity of NMR data (Table 1 and Figure 2) between **3** and **4** indicated that they have the same picrotoxane-type sesquiterpene skeleton. The only structural difference between them was CH-9 (δ_C/H_ 55.2/2.77) in **3** was oxygenated to a hydroxylated quaternary carbon (δ_C_ 85.1) in **4**, as supported by sequential COSY correlations of H-2/H-3/H-4/H-5/H-6/H-7/H-8, together with the HMBC correlations from H-2, H-6, H-7, and H_3_-10 to C-9. The stereochemical structure of **4** (1*S*, 2*S*, 3*R*, 4*S*, 5*R*, 6*S*, and 9*R*), was determined by a single-crystal X-ray diffraction pattern obtained using the anomalous scattering of Cu Kα radiation (Figure 4). From a biosynthetic consideration, the absolute configurations of **4** were deduced be identical to those of **1**-**3**. Hence, compound **4** was also identified as a picrotoxane sesquiterpene as shown in Figure 1, named as dendroterpene D.

### The Postulated Biosynthesis Pathway of Compounds 1–4

The carbon skeletons of previously reported picrotoxane-type sesquiterpenes are highly conserved.

**Figure 7:**
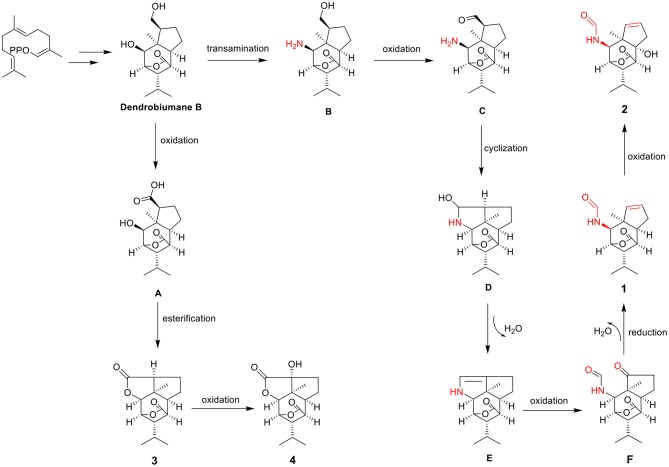
Hypothetical biogenetic pathway of compounds **1**–**4**.

The main skeletal changes are that one is the C-11-O-C-2 ring or the C-11-N-C-2 ring closure exemplified by dendrobiumane E and the other is the C-11-O-C-2 ring or the C-11-N-C-2 ring opening illustrated by dedrobiumane B. In our present study, the discovery of **1** and **2** with a new carbon skeleton containing a formamide group, which was derived from unprecedented carbon bond cleavage pattern prompted us to study its plausible biosynthetic pathways (Figure 7). Dendrobiumane B was assumed to be the biosynthetic precursor of **1**–**4**. Dendrobiumane **B** underwent oxidation to produce the intermediate (**A**). **A** underwent esterification and the C-11-O-C-2 ring closure produced compound **3**, which underwent oxidation to afford compound **4**. Dendrobiumane **B** underwent transamination to give the intermediate (**B**), which underwent oxidation to form intermediate (**C**). **C** underwent cyclization to produce intermediate (**D**), and **D** underwent dehydration to yield intermediate (**E**). **E** underwent oxidation to give intermediate (**F**), which underwent reduction and dehydration to produce compound **1**. Then, **1** underwent oxidation to get **2**.

### Biological Activity

Compounds **1**–**8** were tested for inhibitory activity against α-glycosidase using the PNPG method and for cytotoxic effects on SGC-7901, K562, A549, BEL-7402, and Hela cell lines using the MTT method (See Supplementary Table 1 in the Supplementary Material). The result showed that compounds **3**, **5**, **6**, and **8** exhibited potent inhibitory activity against α-glycosidase with IC_50_ values of 0.97, 0.03, 0.68, and 0.30 mM, respectively (Acarbose as positive control with IC_50_ value of 0.72 mM). Compound **5** displayed weak cytotoxic effects against SGC-7901, K562, A549, BEL-7402, and Hela cell lines with the IC_50_ values of 17.30, 10.39, 29.03, 20.13, and 22.19 μM, respectively (Cisplatin as positive control with IC_50_ values of 4.11, 3.08, 1.93, 4.02, and 11.29 μM), in addition, compound **6** showed cytotoxic effects against K562 with the IC_50_ value of 28.23 μM.

## Conclusions

In conclusion, four new picrotoxane-type sesquiterpenes (**1**–**4**), together with four known compounds (**5**–**8**), were isolated from the stems of *D. nobile*. Compounds **1** and **2** are two new picrotoxane-type sesquiterpenes with a new carbon skeleton containing a formamide group, which may be derived from the previously reported dendrobiumane B skeleton by the C(9)-C(11) carbon bond cleavage. In the previously report, picrotoxane sesquiterpenes exhibited the angiogenesis effect against sunitinibinduced damage (Meng et al., 2017) and inhibitory activity of nerve growth factor mediated neurite outgrowth (Leon et al., 2019). Compound **3** exhibited inhibitory activity against α-glycosidase with IC_50_ values of 0.97 mM. However, compound **4** is inactive (IC_50_ > 1 mM) against α-glycosidase, indicating that the hydroxyl at C-9 may reduce the activity. In addition, the known bibenzyl derivative nobilin E (**5**) exhibited inhibitory effects on NO production in activated murine macrophage-like cell line RAW 264.7 in the previously report (Zhang et al., 2007b). The bioactivities of the known bibenzyl derivative dendrocandin V (**6**) have not been reported. In this report, compounds **5** and **6** both exhibited inhibitory activity against α-glycosidase with IC_50_ values of 0.03 and 0.68 mM, respectively. In addition, Compound **5** displayed weak cytotoxic effects against SGC-7901, K562, A549, BEL-7402 and Hela cell lines with the IC_50_ values of 17.30, 10.39, 29.03, 20.13, and 22.19 μM, respectively. Compound **6** showed cytotoxic effect against K562 with the IC_50_ value of 28.23 μM.

## Data Availability Statement

All datasets generated for this study are included in the article/Supplementary Material.

## Author Contributions

PW, XC, HW, CC, JY, and XX performed the experiments. PW and XC identified the structures. GZ performed ECD calculation. PW, HD, and WM conceived and designed the experiments. SH collected the fresh stems of *D. nobile*. PW wrote the paper. All authors have approved the final version of the manuscript.

### Conflict of Interest

The authors declare that the research was conducted in the absence of any commercial or financial relationships that could be construed as a potential conflict of interest.

## Supplementary Material

These data include 1D and 2D NMR spectra of compounds **1**–**4**, CD spectra of **1** and **2**, and cytotoxic and α-glycosidase inhibitory activities of compounds **1**–**8**.
