# Supplementary material for: Four New Picrotoxane-Type Sesquiterpenes From Dendrobium nobile Lindl
Source: Front Chem. 2019 Nov 29;7:812. doi: 10.3389/fchem.2019.00812 (PMC6895213; doi:10.3389/fchem.2019.00812)
Supplement: Supplementary file 1 [file Data_Sheet_1.docx]

**Supplementary Material**

Table of Contents

**Supplementary Figure 1.** The ^1^H NMR (500 MHz, CD_3_OH-*d*_4_) spectrum of **1**

**Supplementary Figure 2.** The ^13^C NMR and DEPT135 (125 MHz, CD_3_OH-*d*_4_) spectra of **1**

**Supplementary Figure 3.** The ^1^H-^1^H COSY (500 MHz, CD_3_OH-*d*_4_) spectrum of **1**

**Supplementary Figure 4.** The HSQC (500 MHz, CD_3_OH-*d*_4_) spectrum of **1**

**Supplementary Figure 5.** The HMBC (500 MHz, CD_3_OH-*d*_4_) spectrum of **1**

**Supplementary Figure 6.** The ROESY (500 MHz, CD_3_OH-*d*_4_) spectrum of **1**

**Supplementary Figure 7.** The ^1^H NMR (500 MHz, DMSO-*d*_6_) spectrum of **2**

**Supplementary Figure 8.** The ^13^C NMR and DEPT135 (125 MHz, DMSO-*d*_6_) spectra of **2**

**Supplementary Figure 9.** The ^1^H-^1^H COSY (500 MHz, DMSO-*d*_6_) spectrum of **2**

**Supplementary Figure 10.** The HSQC (500 MHz, DMSO-*d*_6_) spectrum of **2**

**Supplementary Figure 11.** The HMBC (500 MHz, DMSO-*d*_6_) spectrum of **2**

**Supplementary Figure 12.** The ROESY (500 MHz, DMSO-*d*_6_) spectrum of **2**

**Supplementary Figure 13.** The ^1^H NMR (500 MHz, CDCl_3_-*d*) spectrum of **3**

**Supplementary Figure 14.** The ^13^C NMR and DEPT135 (125 MHz, CDCl_3_-*d*) spectra of **3**

**Supplementary Figure 15.** The ^1^H-^1^H COSY (500 MHz, CDCl_3_-*d*) spectrum of **3**

**Supplementary Figure 16.** The HSQC (500 MHz, CDCl_3_-*d*) spectrum of **3**

**Supplementary Figure 17.** The HMBC (500 MHz, CDCl_3_-*d*) spectrum of **3**

**Supplementary Figure 18.** The ROESY (500 MHz, CDCl_3_-*d*) spectrum of **3**

**Supplementary Figure 19.** The ^1^H NMR (500 MHz, CDCl_3_-*d*) spectrum of **4**

**Supplementary Figure 20.** The ^13^C NMR and DEPT135 (125 MHz, CDCl_3_-*d*) spectra of compound **4**

**Supplementary Figure 21.** The ^1^H-^1^H COSY (500 MHz, CDCl_3_-*d*) spectrum of **4**

**Supplementary Figure 22.** The HSQC (500 MHz, CDCl_3_-*d*) spectrum of **4**

**Supplementary Figure 23.** The HMBC (500 MHz, CDCl_3_-*d*) spectrum of **4**

**Supplementary Figure 24**. Measured ECD curves of compounds **1** and **2**

**Supplementary Figure 25**. DFT-optimized structures for low-energy conformers of 1*R*, 2*S*, 3*R*, 4*S*, 5*R*, 6*S*, 9*S*-**3** at B3LYP/6-31G (d) level in methanol (PCM).

**Supplementary Table 1.** Cytotoxic andα-glycosidase inhibitory activities of **1**–**8**

**Supplementary Figure 1.** The ^1^H NMR (500 MHz, CD_3_OH-*d*_4_) spectrum of **1**


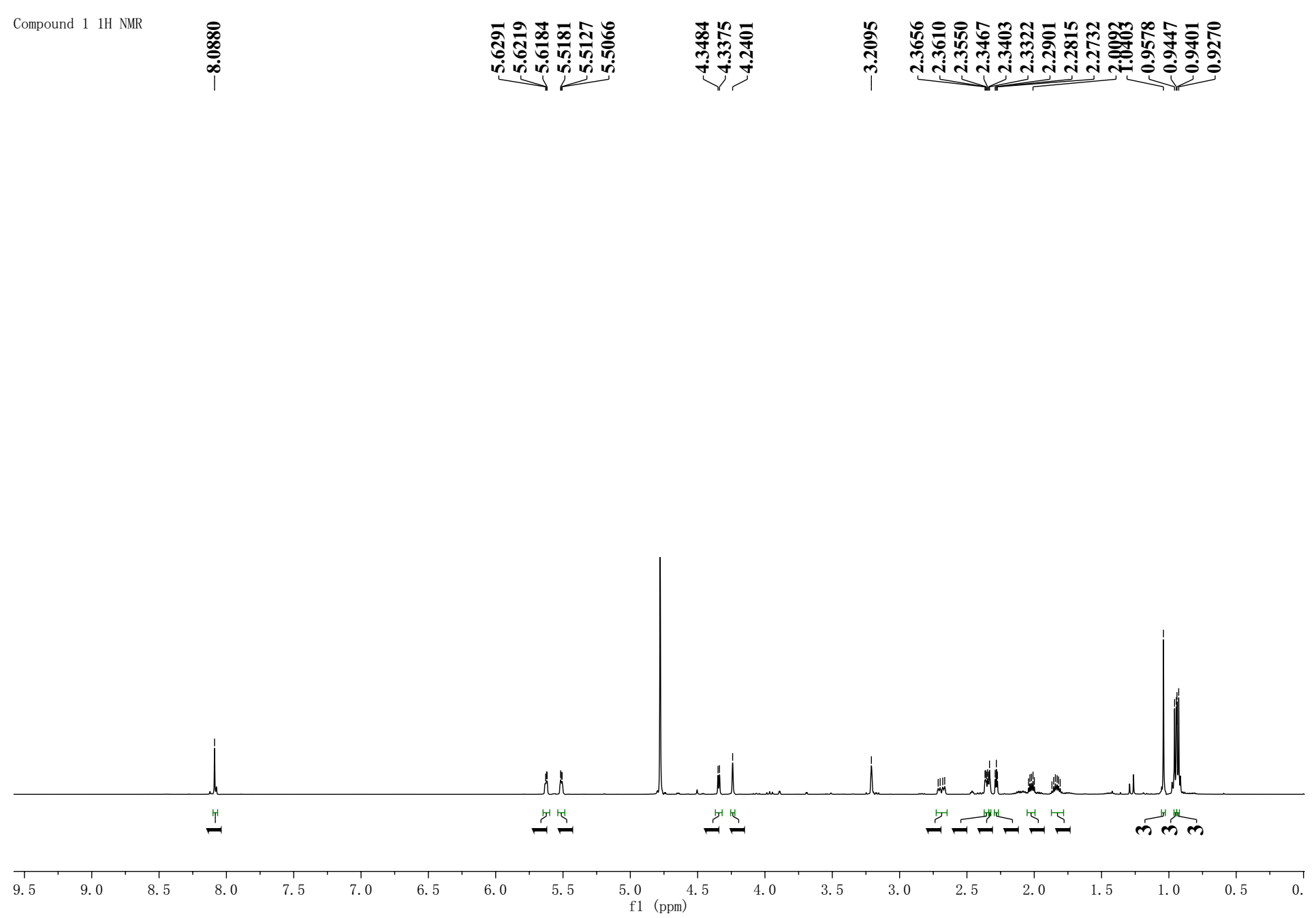


**Supplementary Figure 2.** The ^13^C NMR and DEPT135 (125 MHz, CD_3_OH-*d*_4_) spectra of **1**


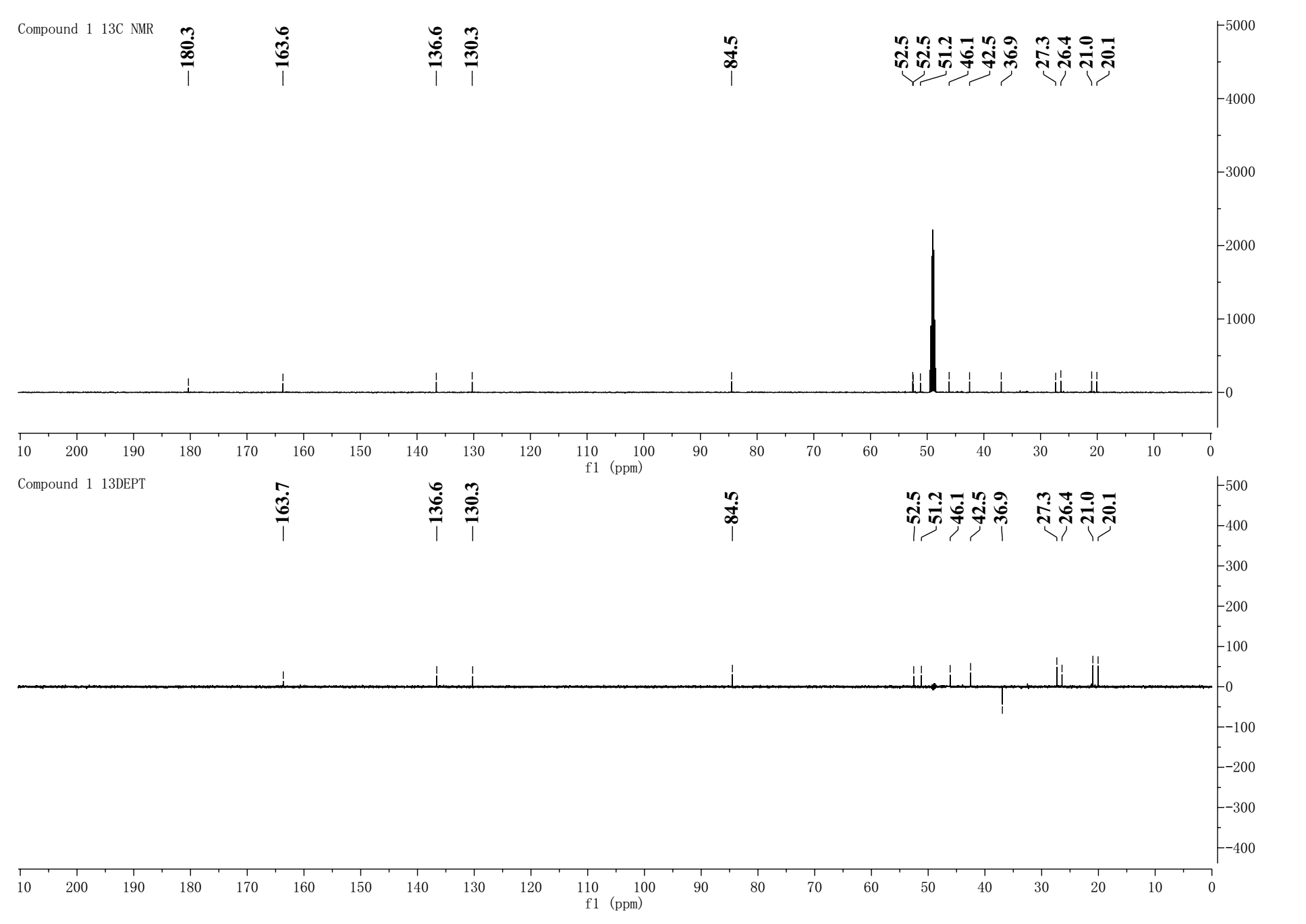


**Supplementary Figure 3.** The ^1^H-^1^H COSY (500 MHz, CD_3_OH-*d*_4_) spectrum of **1**


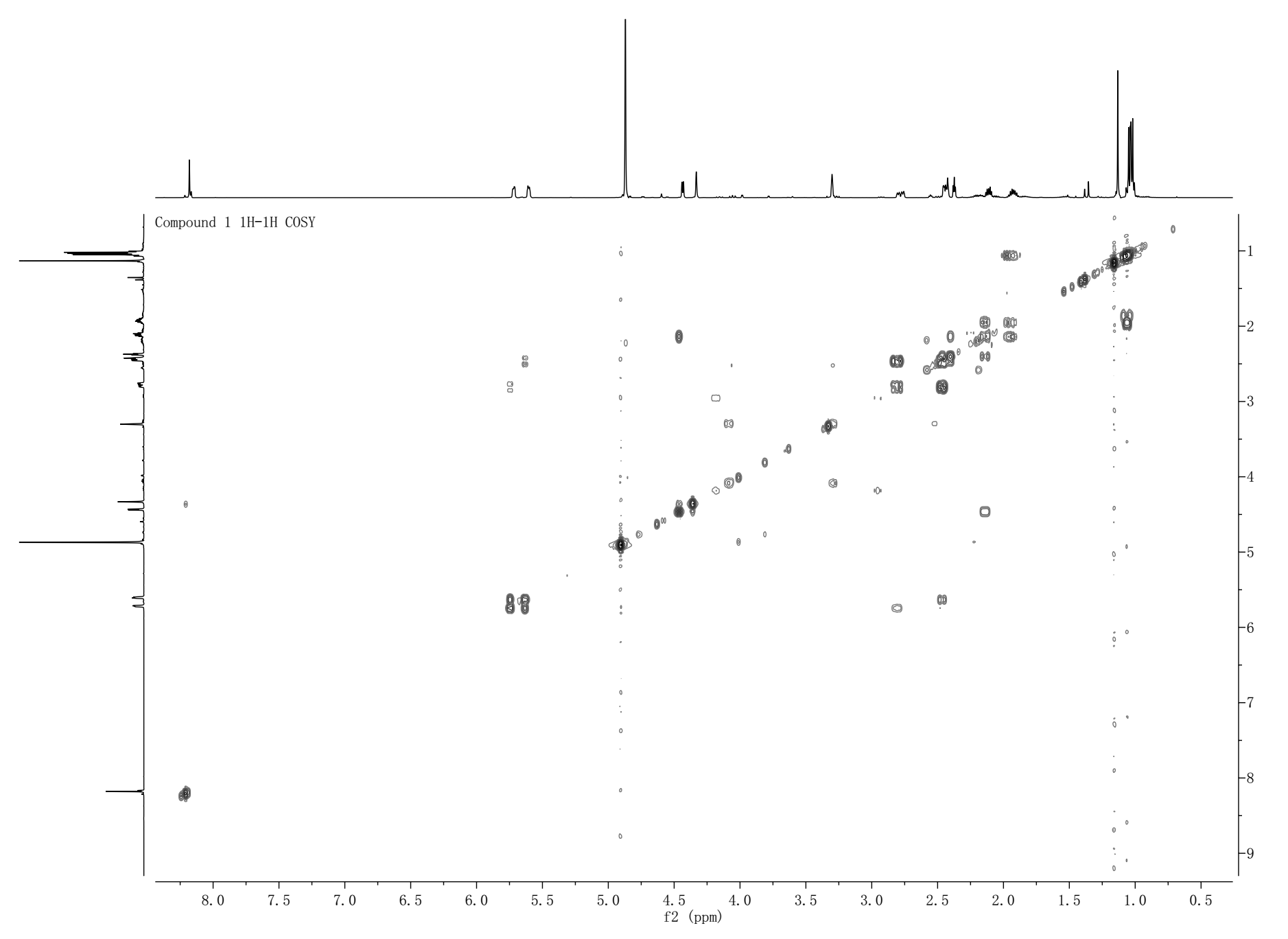


**Supplementary Figure 4.** The HSQC (500 MHz, CD_3_OH-*d*_4_) spectrum of **1**


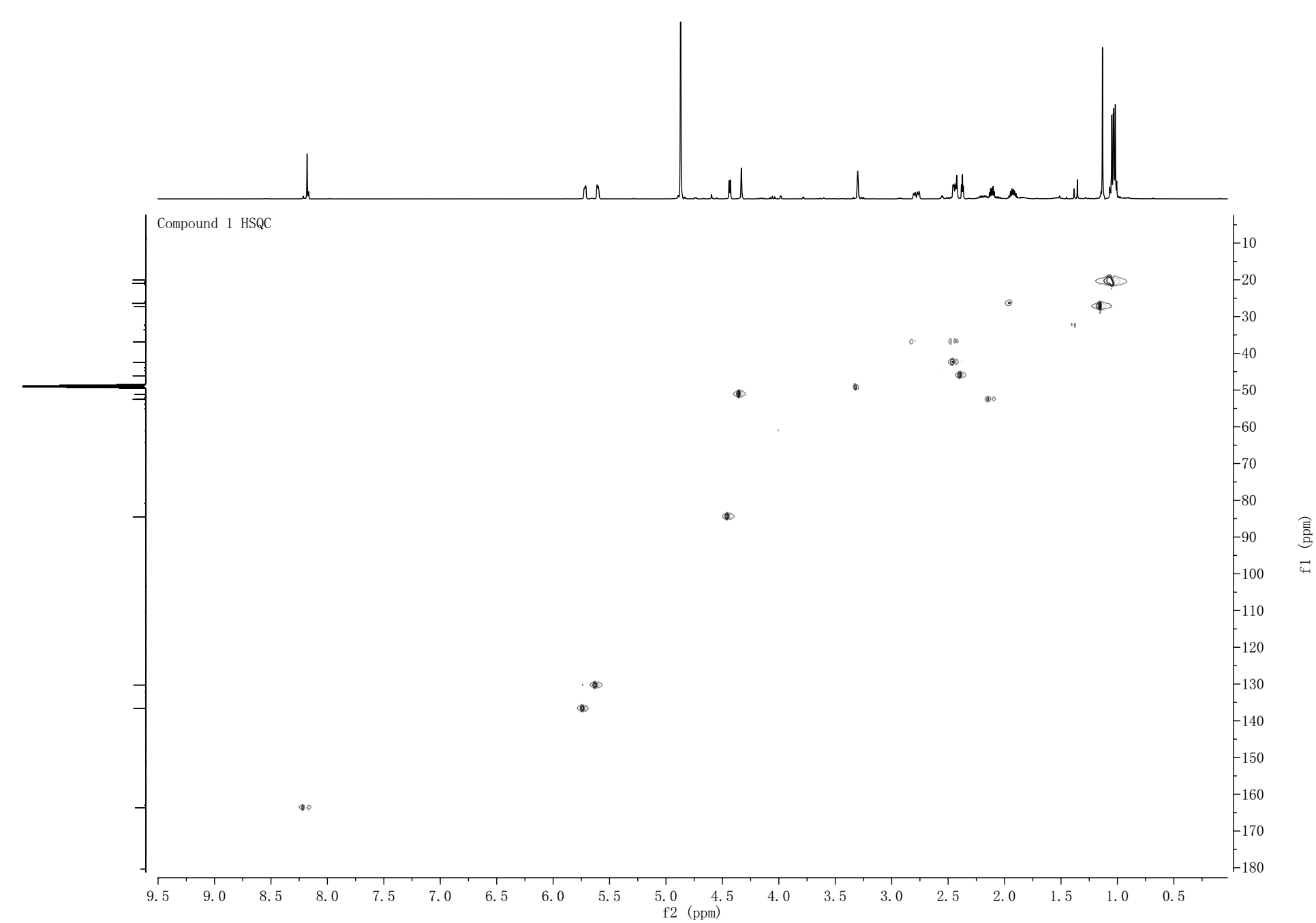


**Supplementary Figure 5.** The HMBC (500 MHz, CD_3_OH-*d*_4_) spectrum of **1**


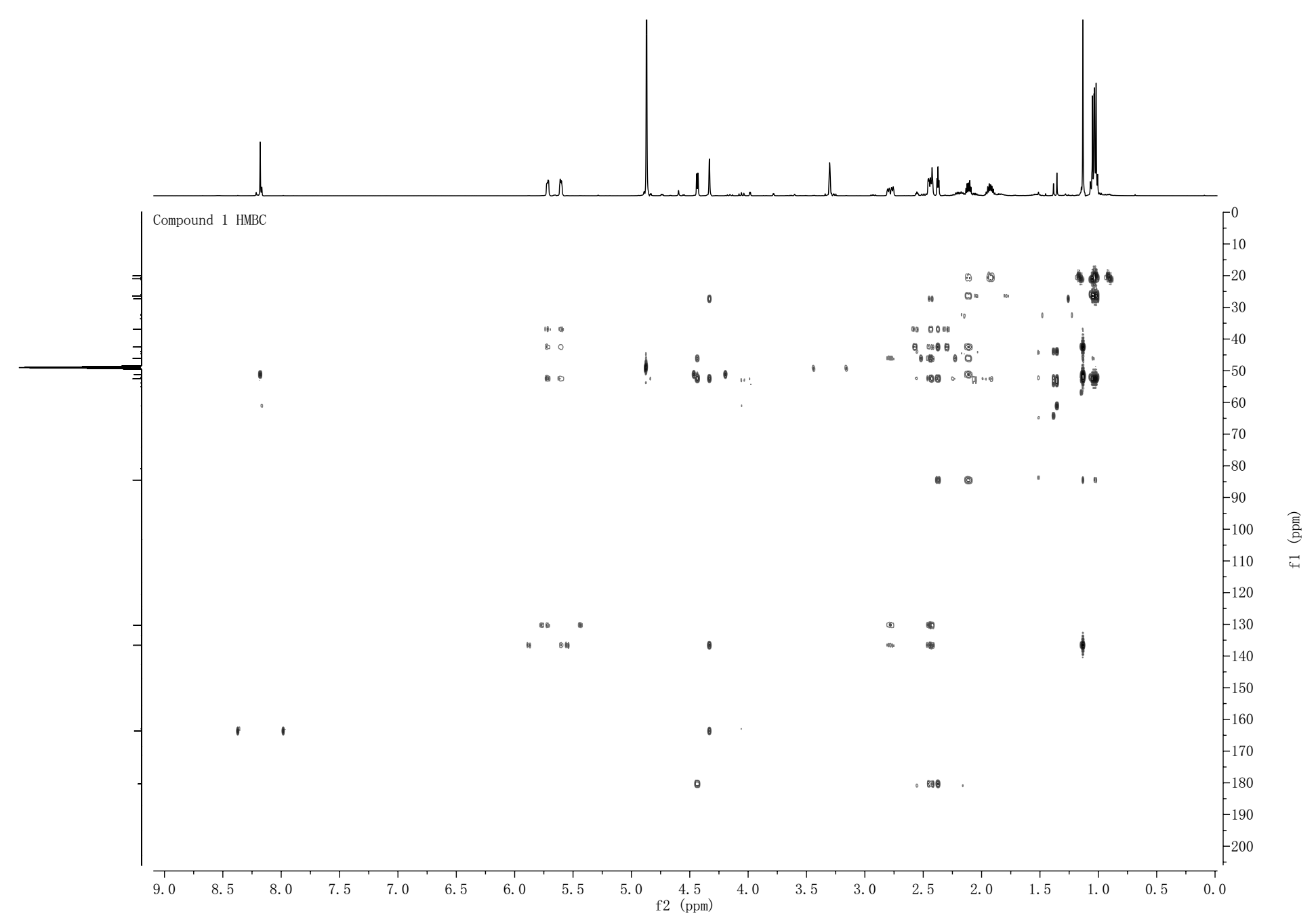


**Supplementary Figure 6.** The ROESY (500 MHz, CD_3_OH-*d*_4_) spectrum of **1**


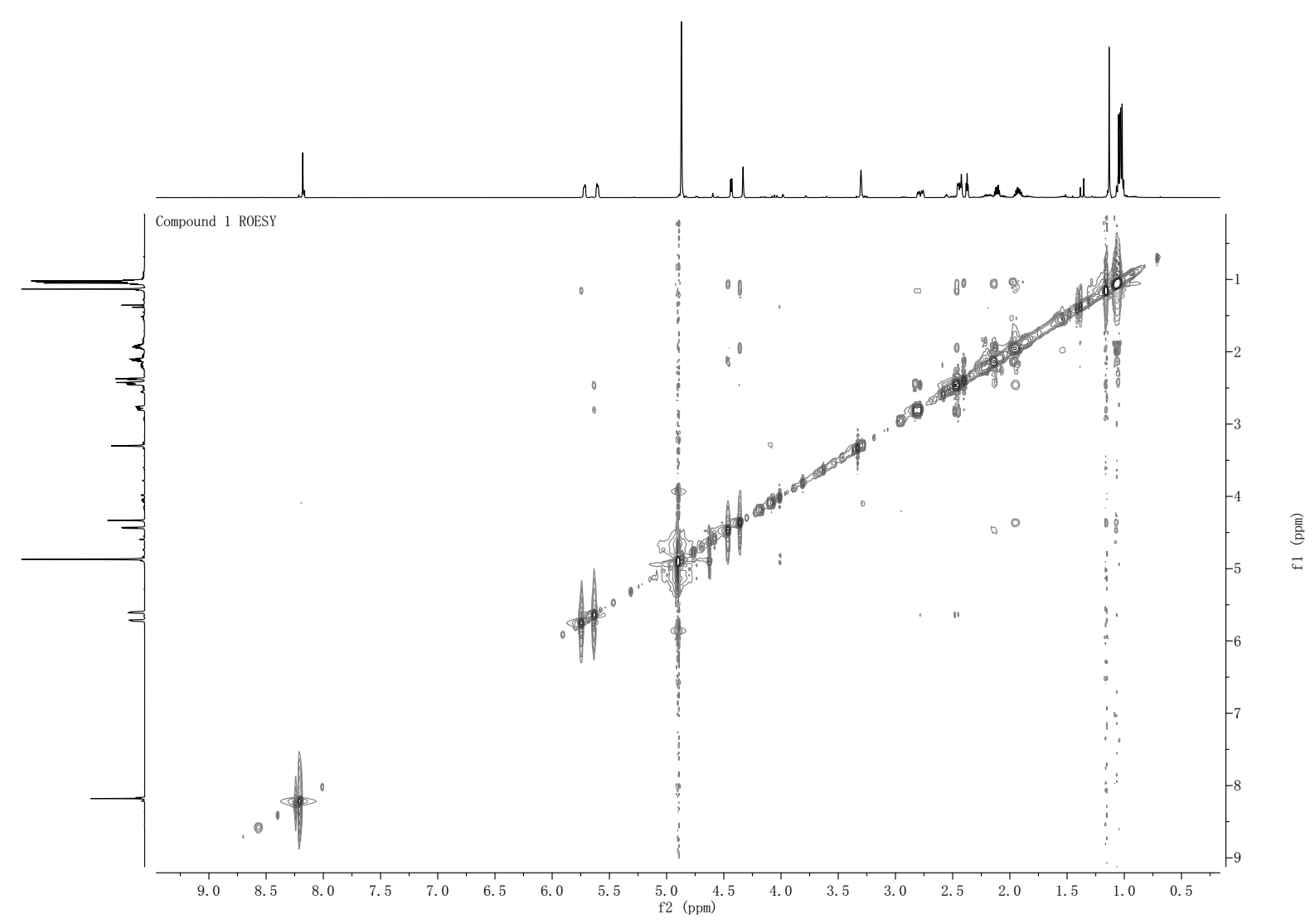


**Supplementary Figure 7.** The ^1^H NMR (500 MHz, DMSO-*d*_6_) spectrum of **2**


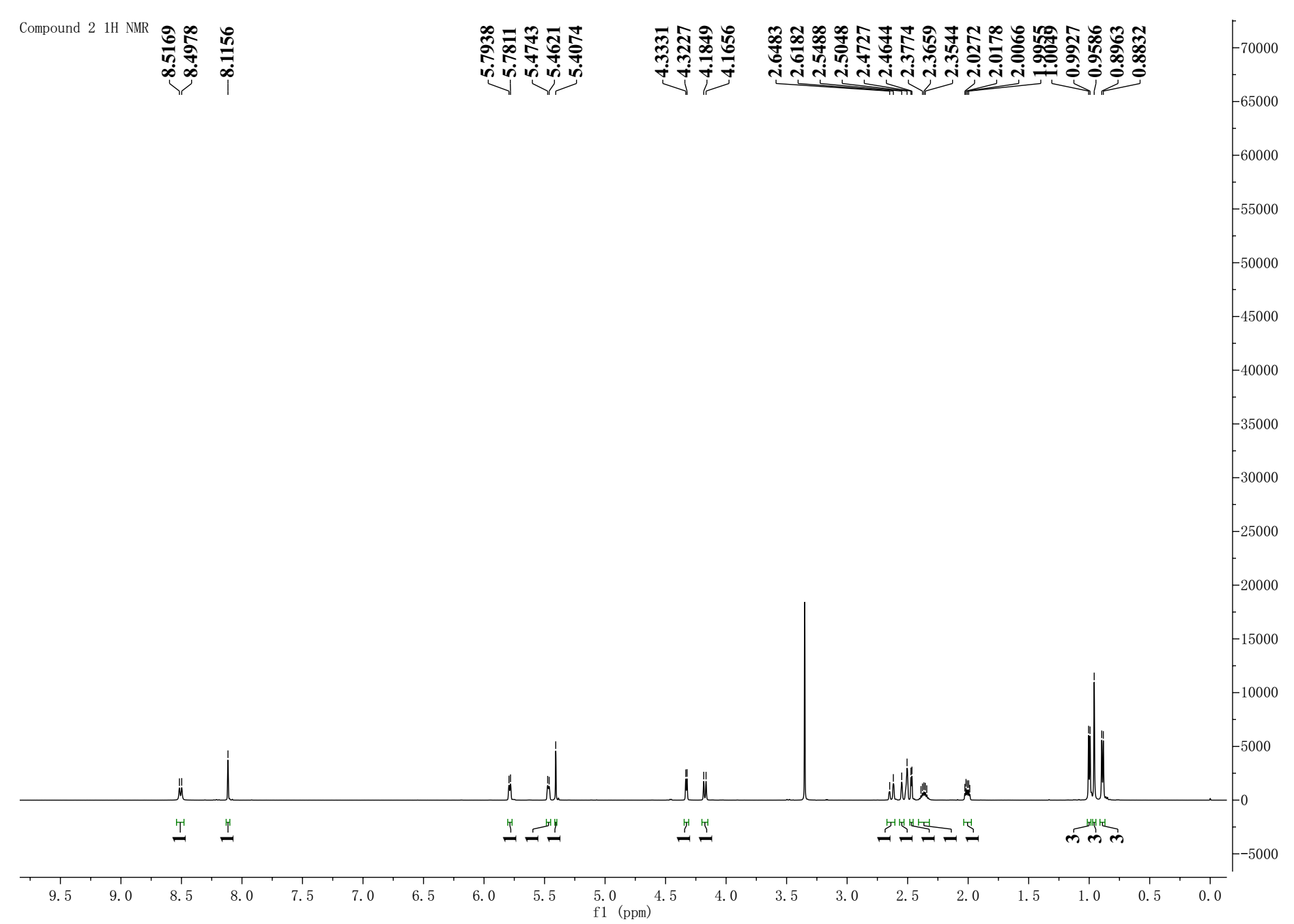


**Supplementary Figure 8.** The ^13^C NMR and DEPT135 (125 MHz, DMSO-*d*_6_) spectra of **2**


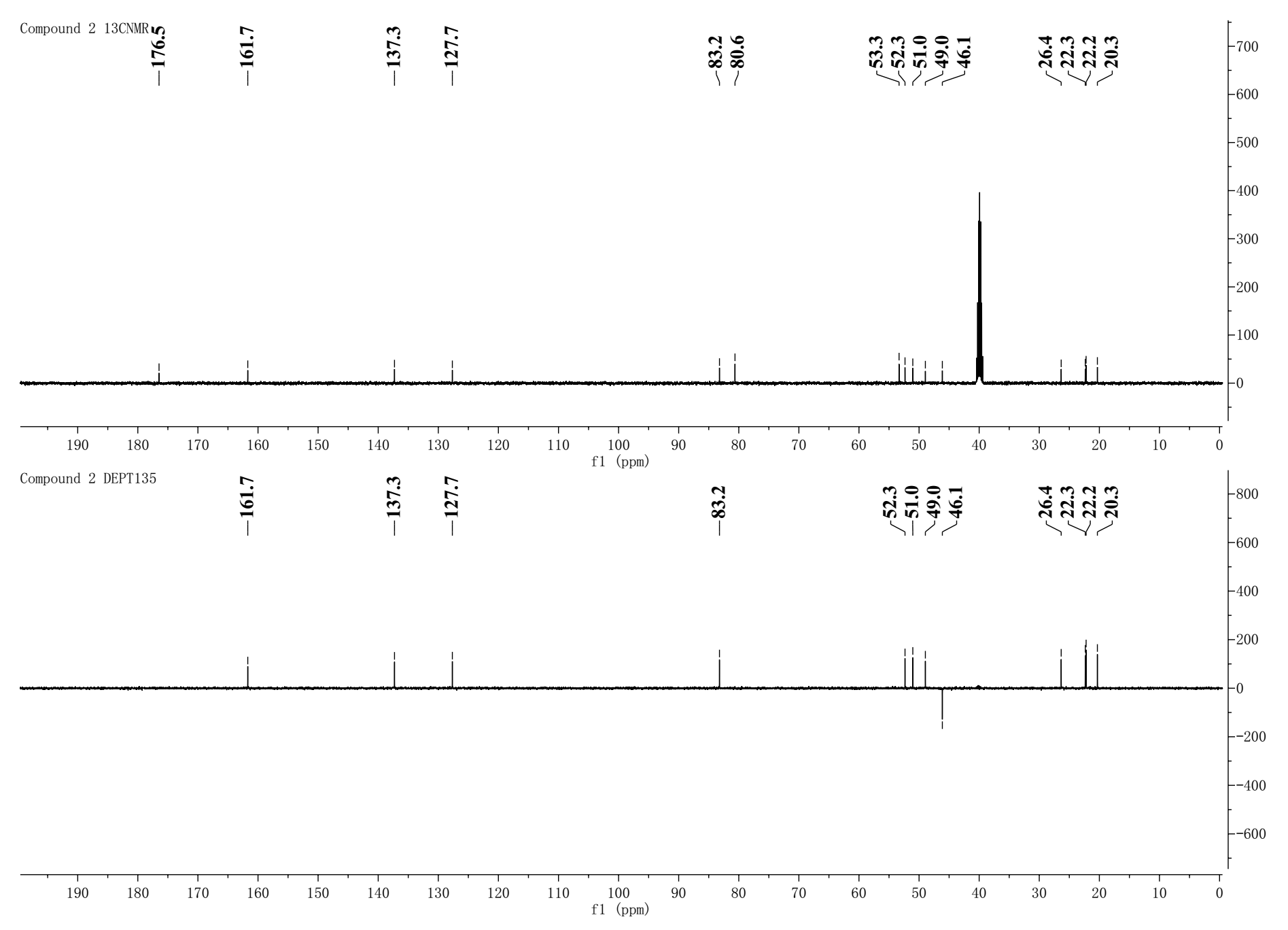


**Supplementary Figure 9.** The ^1^H-^1^H COSY (500 MHz, DMSO-*d*_6_) spectrum of **2**


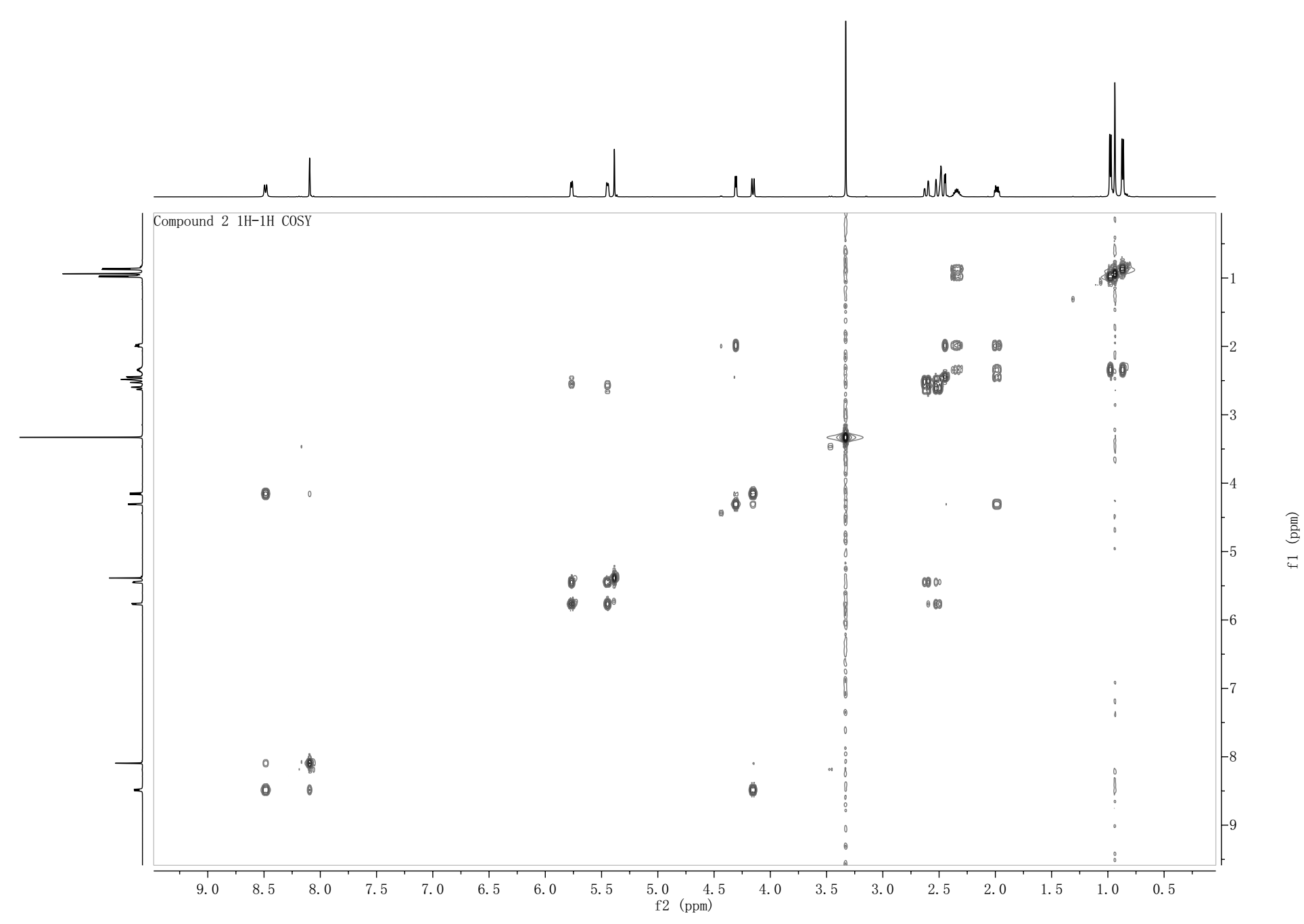


**Supplementary Figure 10.** The HSQC (500 MHz, DMSO-*d*_6_) spectrum of **2**


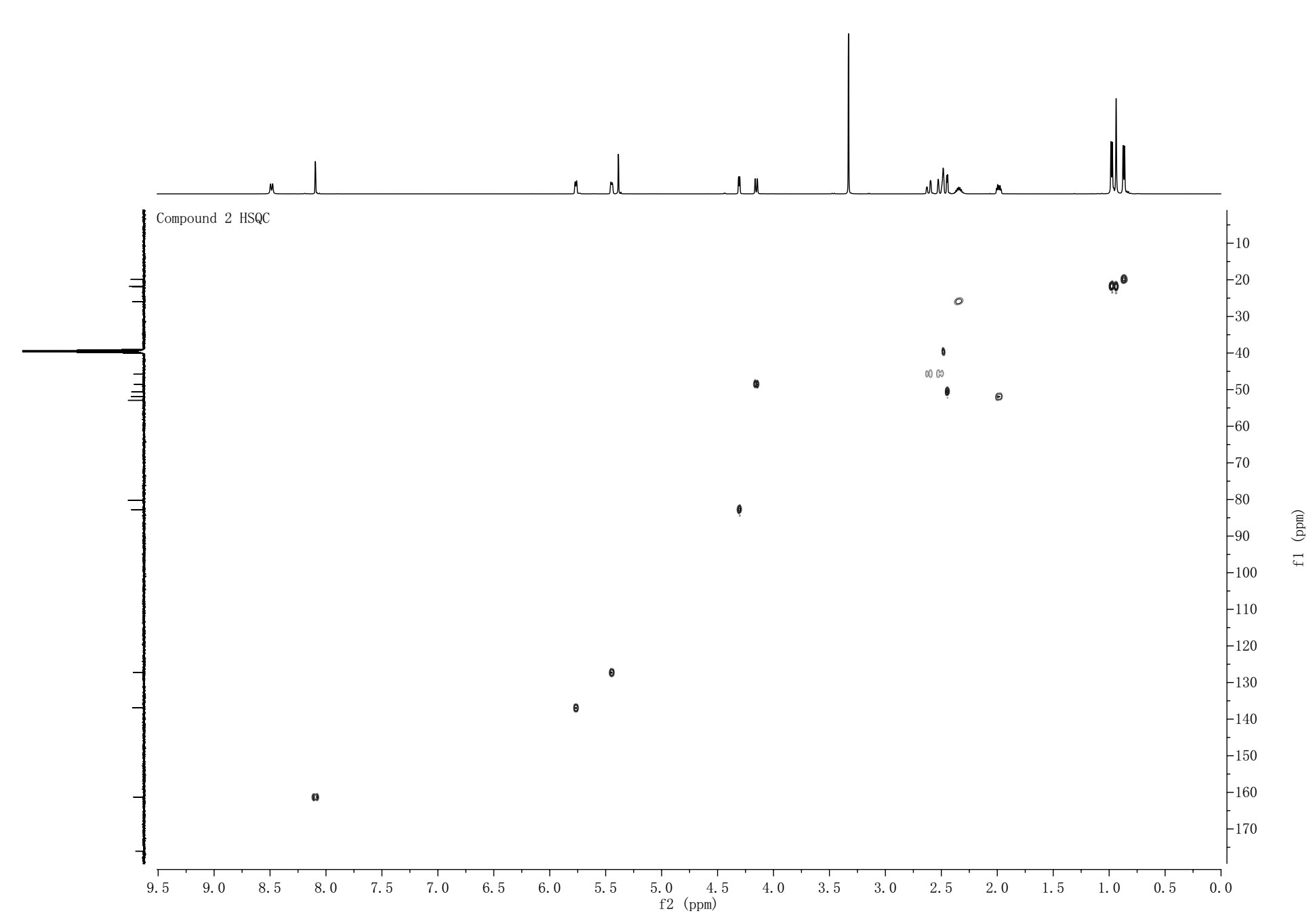


**Supplementary Figure 11.** The HMBC (500 MHz, DMSO-*d*_6_) spectrum of **2**


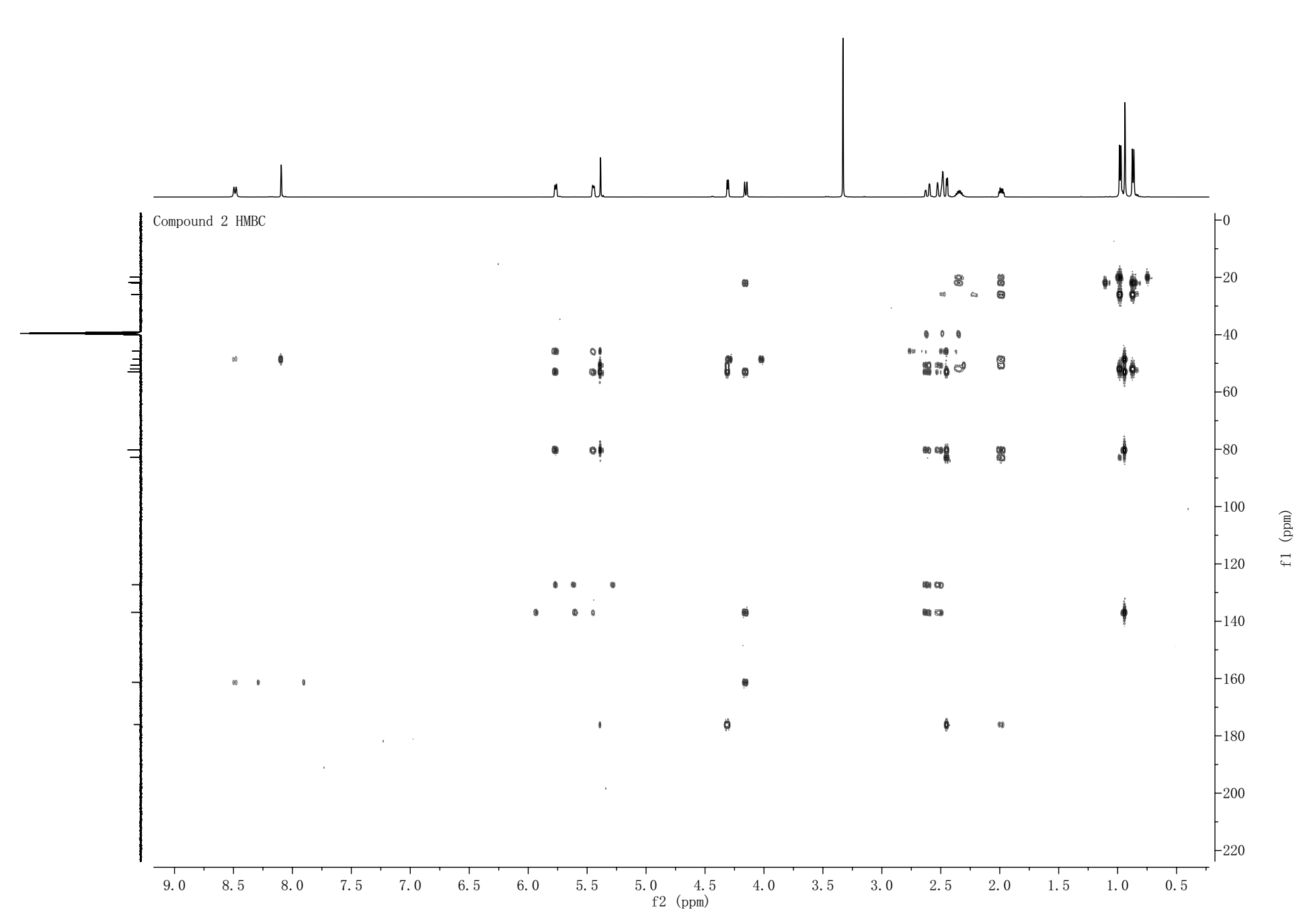


**Supplementary Figure 12.** The ROESY (500 MHz, DMSO-*d*_6_) spectrum of **2**


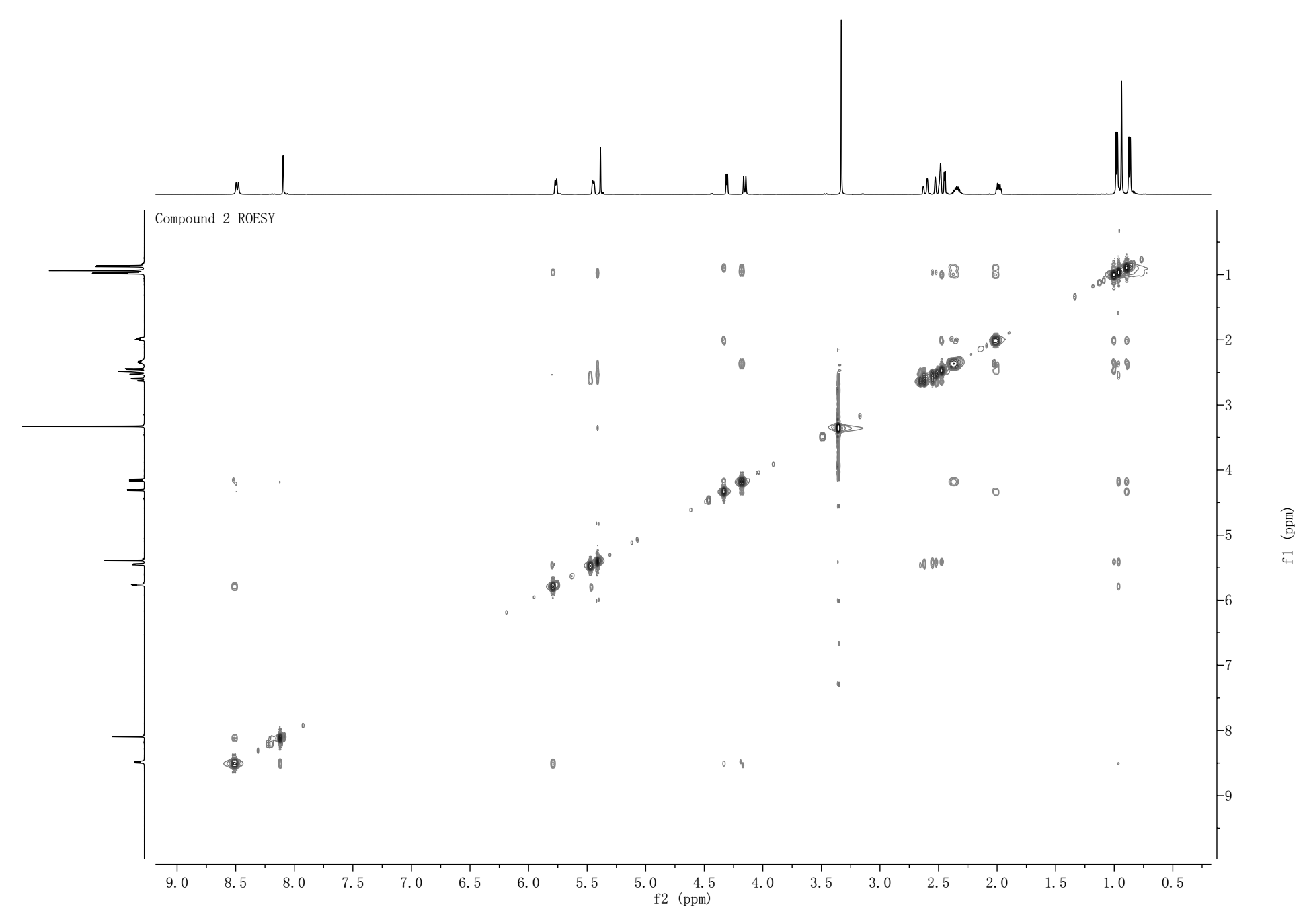


**Supplementary Figure 13.** The ^1^H NMR (500 MHz, CDCl_3_-*d*) spectrum of **3**


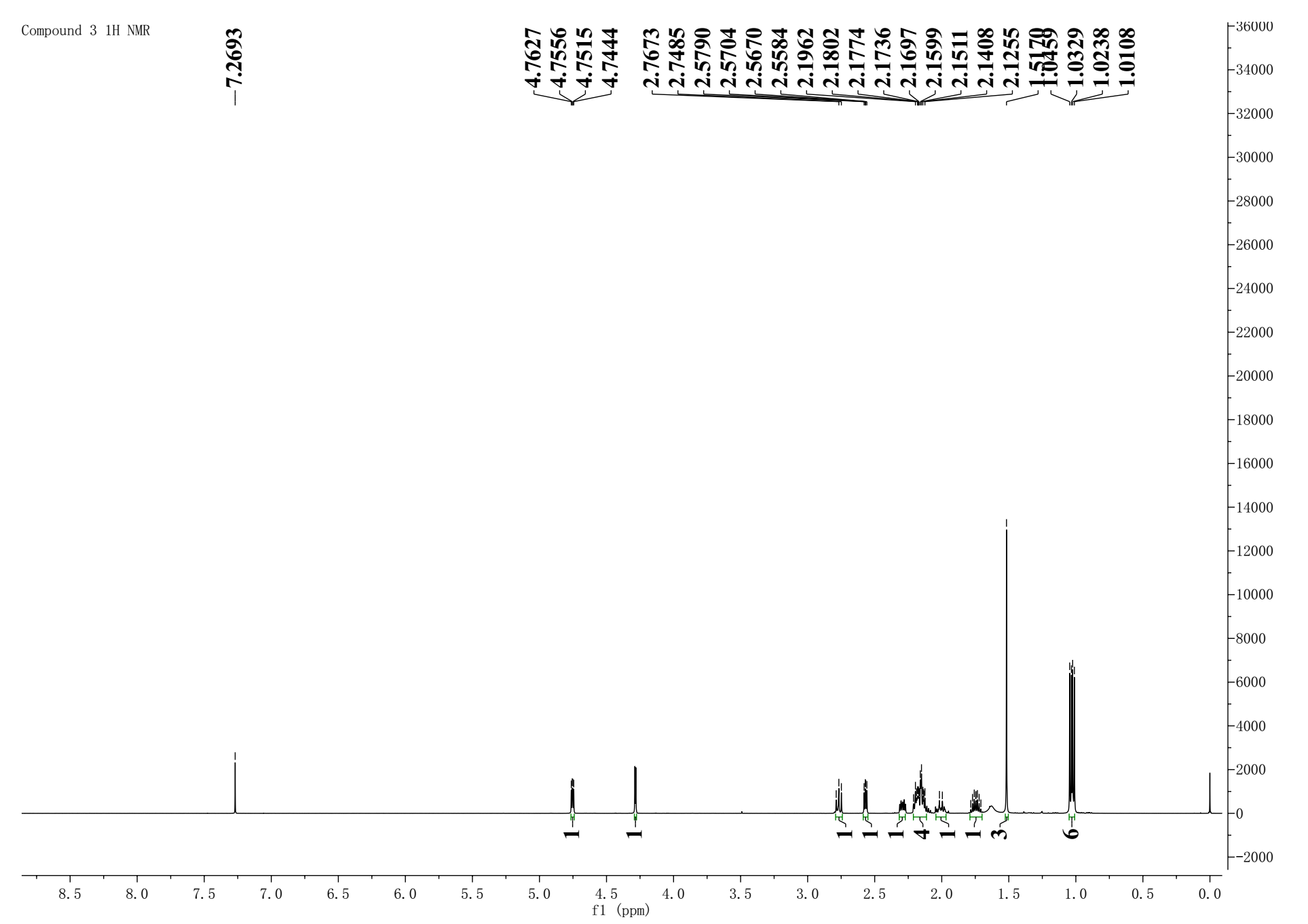


**Supplementary Figure 14.** The ^13^C NMR and DEPT135 (125 MHz, CDCl_3_-*d*) spectra of **3**


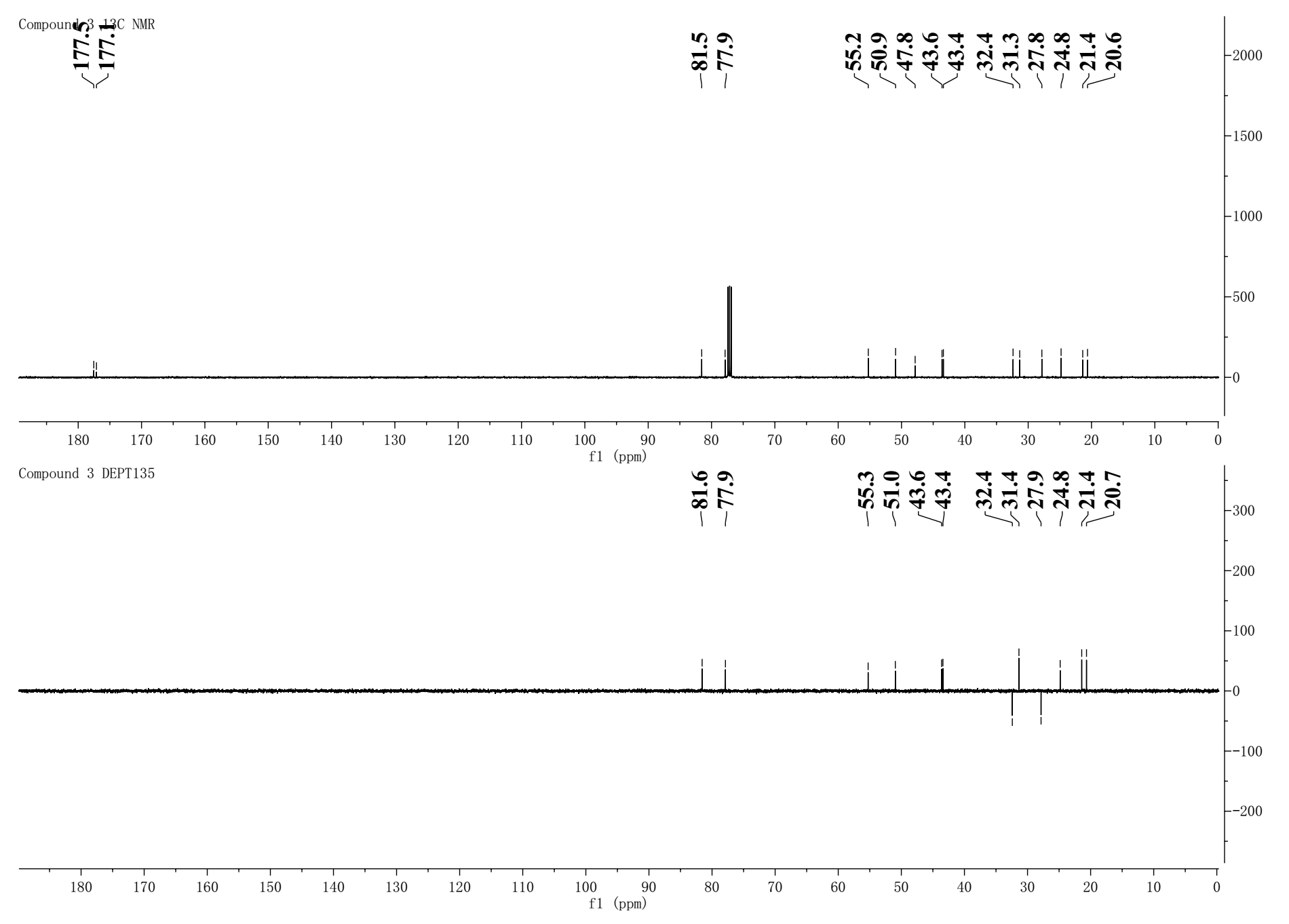


**Supplementary Figure 15.** The ^1^H-^1^H COSY (500 MHz, CDCl_3_-*d*) spectrum of **3**


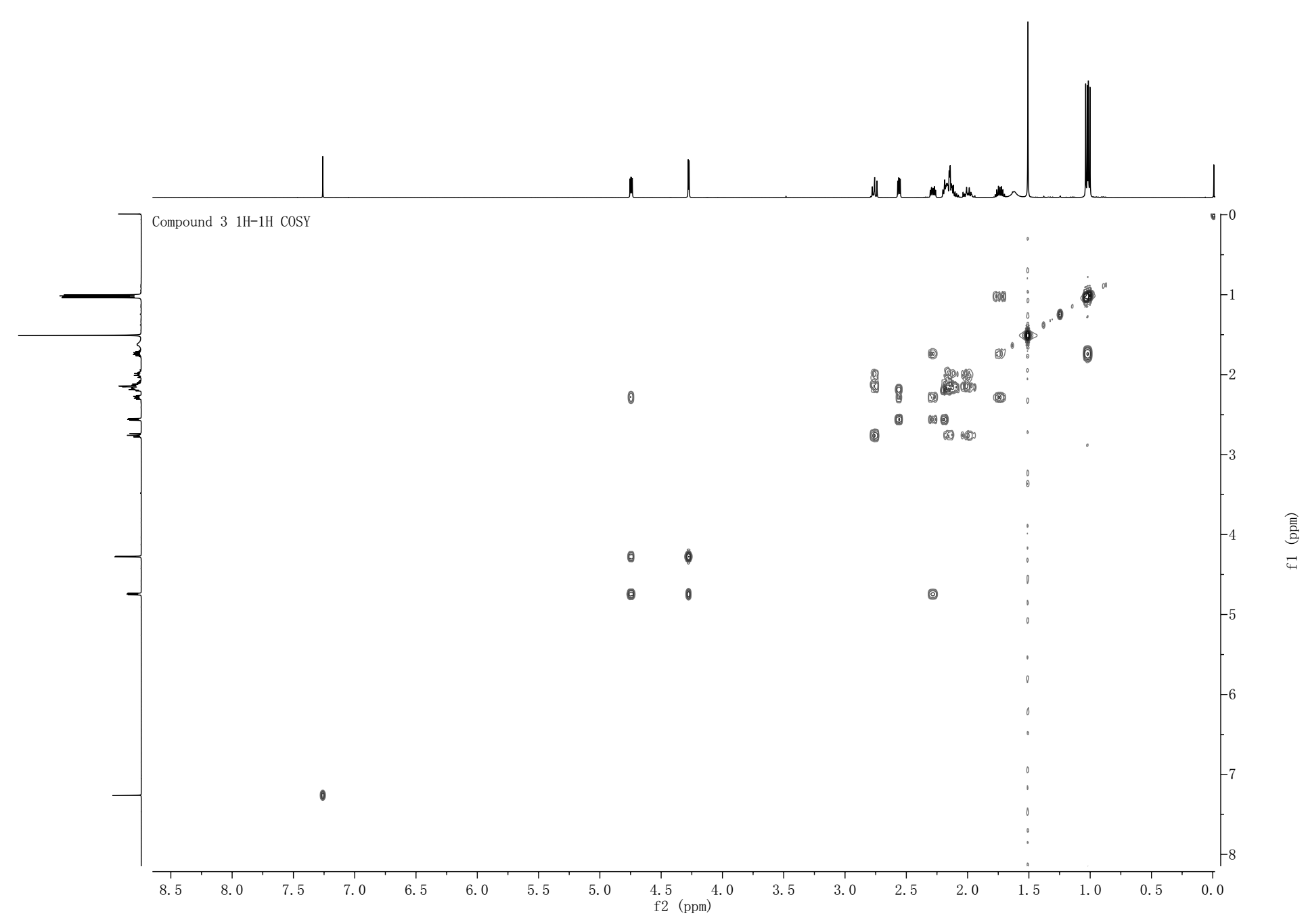


**Supplementary Figure 16.** The HSQC (500 MHz, CDCl_3_-*d*) spectrum of **3**


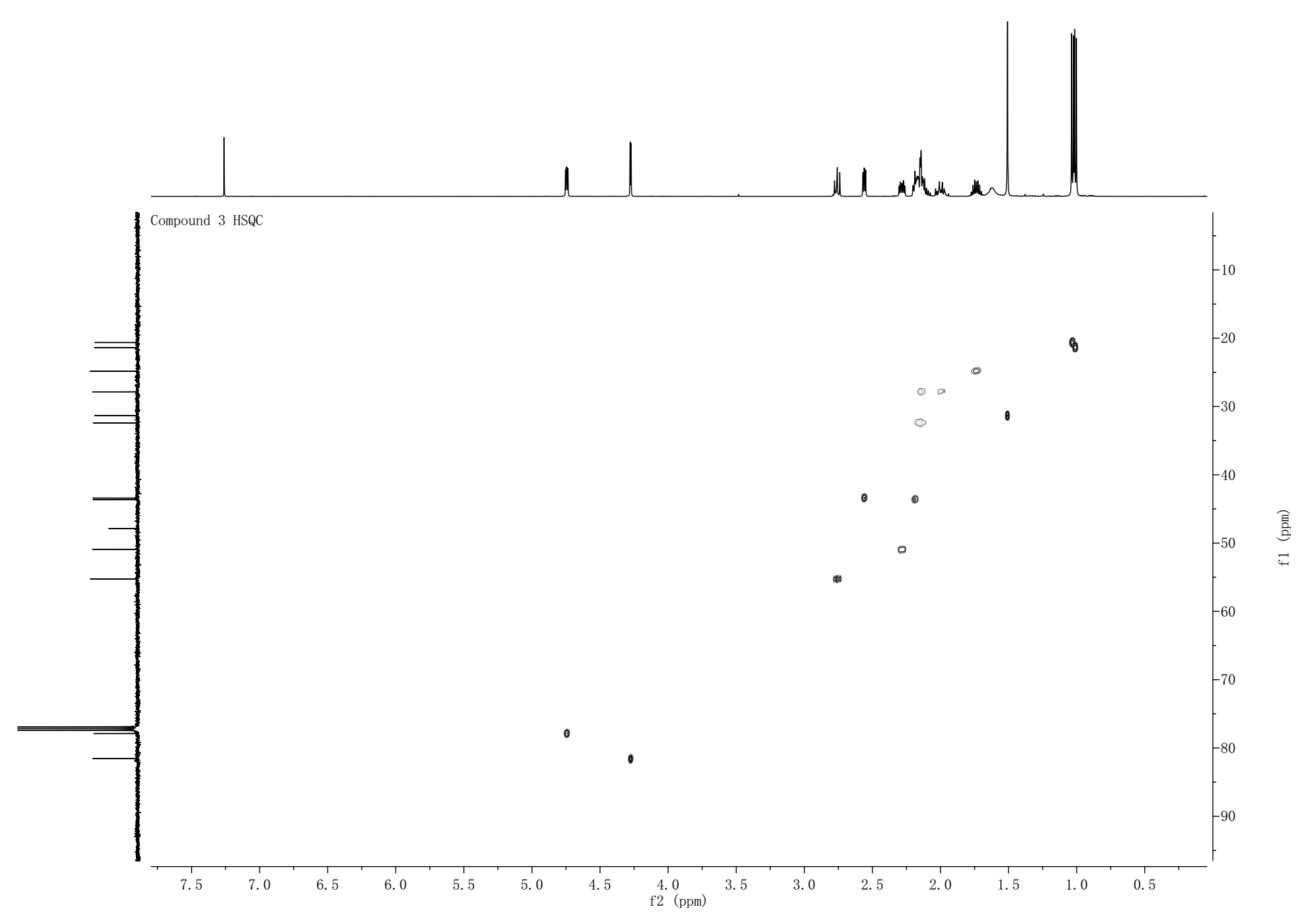


**Supplementary Figure 17.** The HMBC (500 MHz, CDCl_3_-*d*) spectrum of **3**


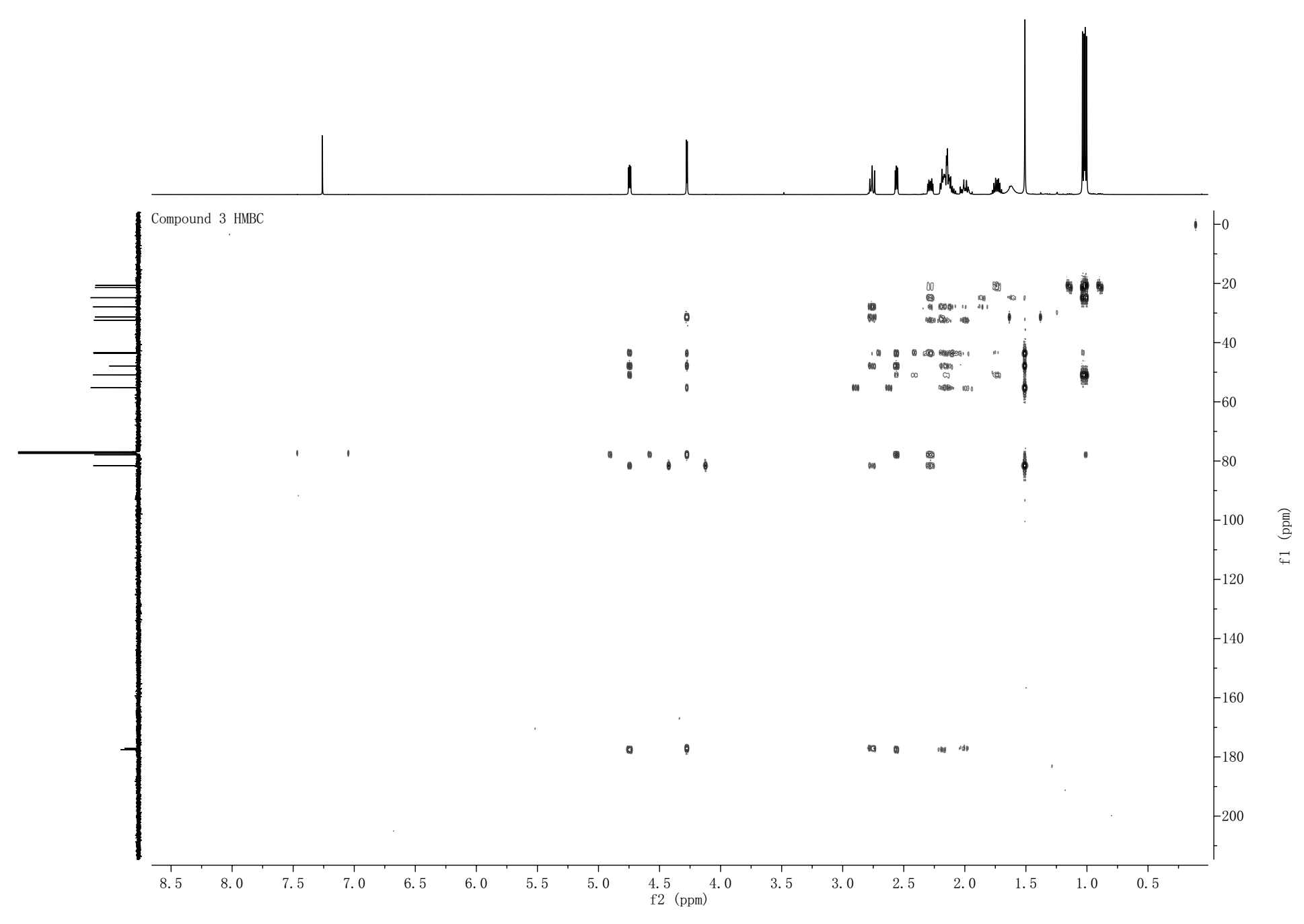


**Supplementary Figure 18.** The ROESY (500 MHz, CDCl_3_-*d*) spectrum of **3**


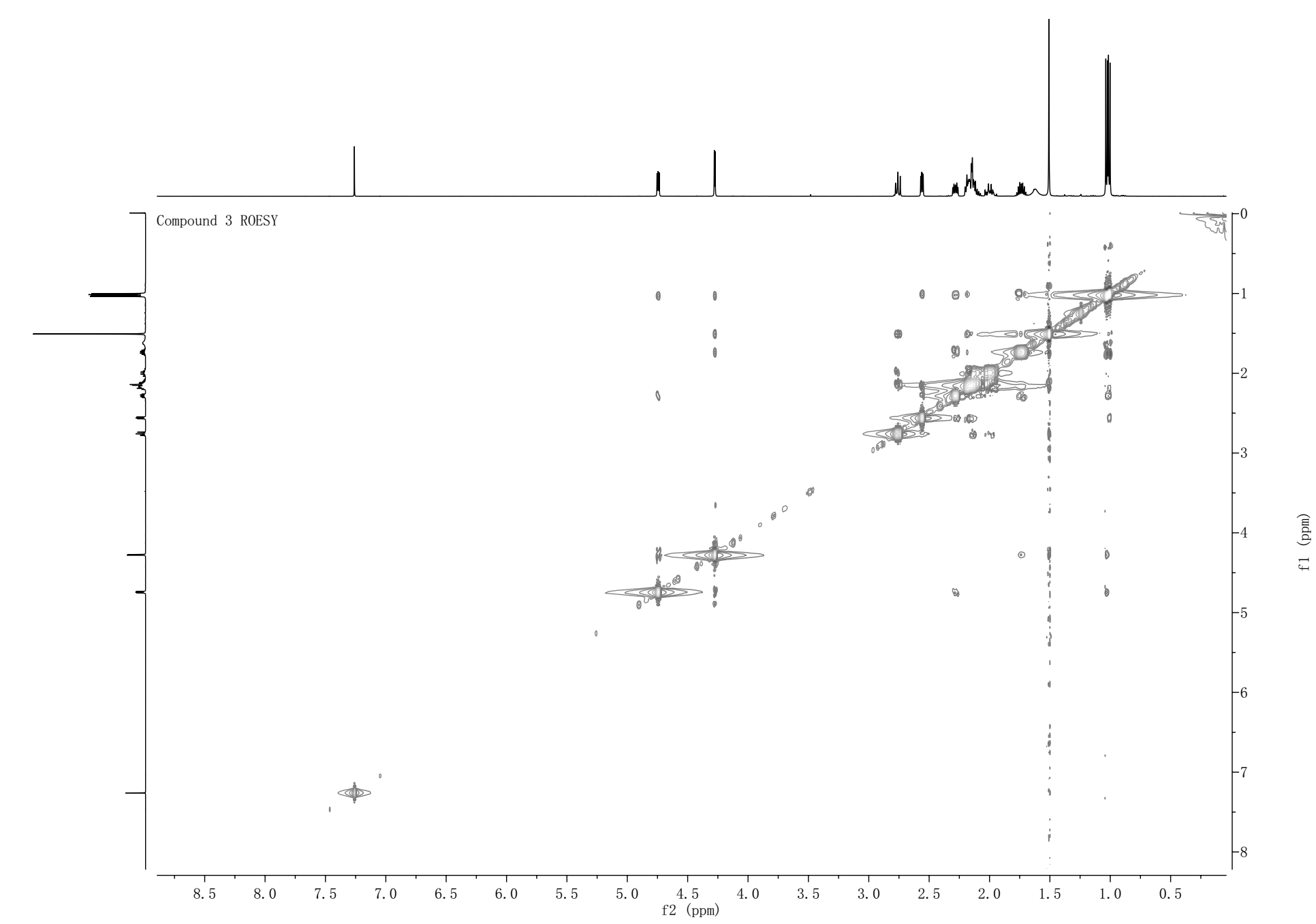


**Supplementary Figure 19.** The ^1^H NMR (500 MHz, CDCl_3_-*d*) spectrum of **4**


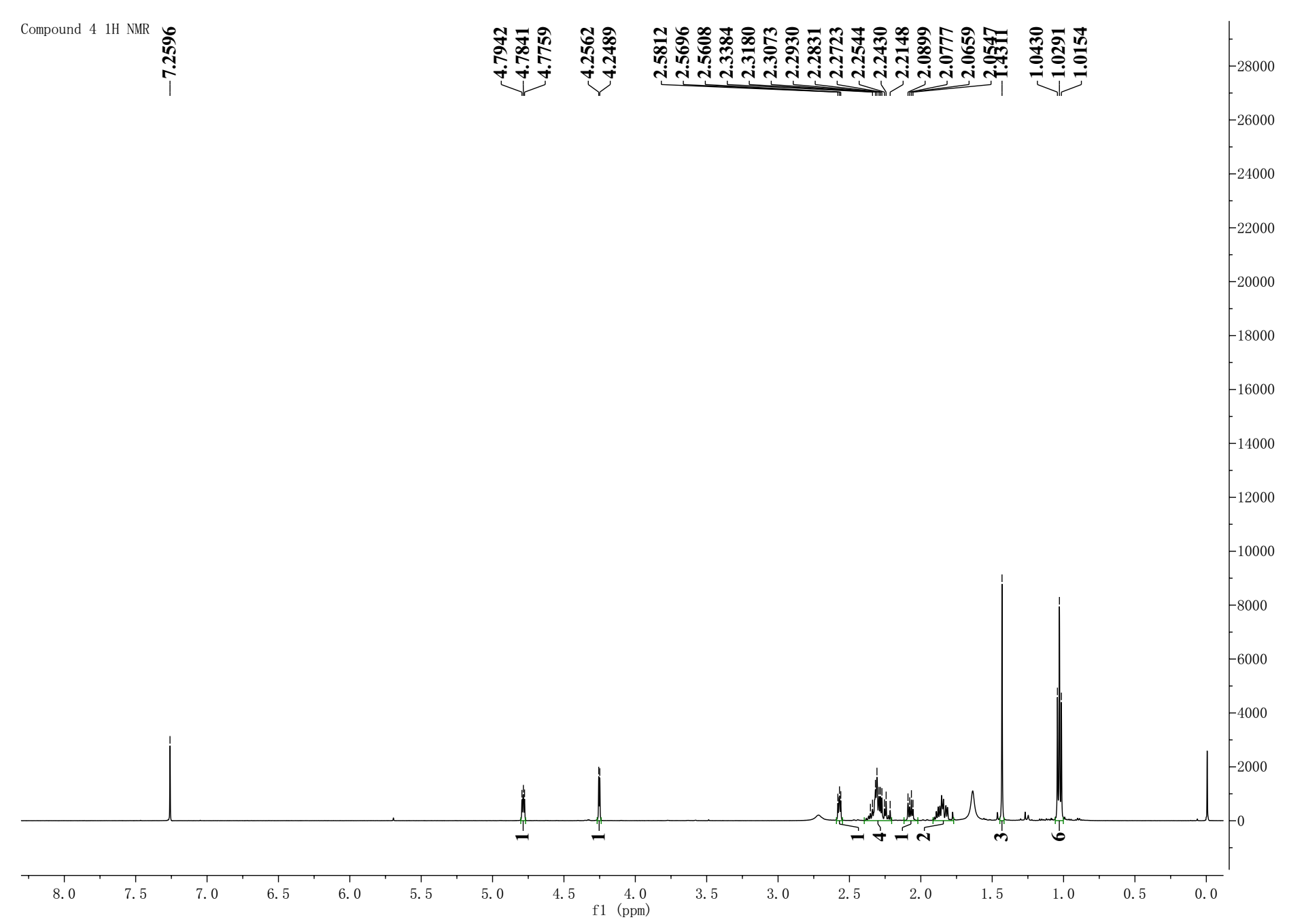


**Supplementary Figure 20.** The ^13^C NMR and DEPT135 (125 MHz, CDCl_3_-*d*) spectra of **4**


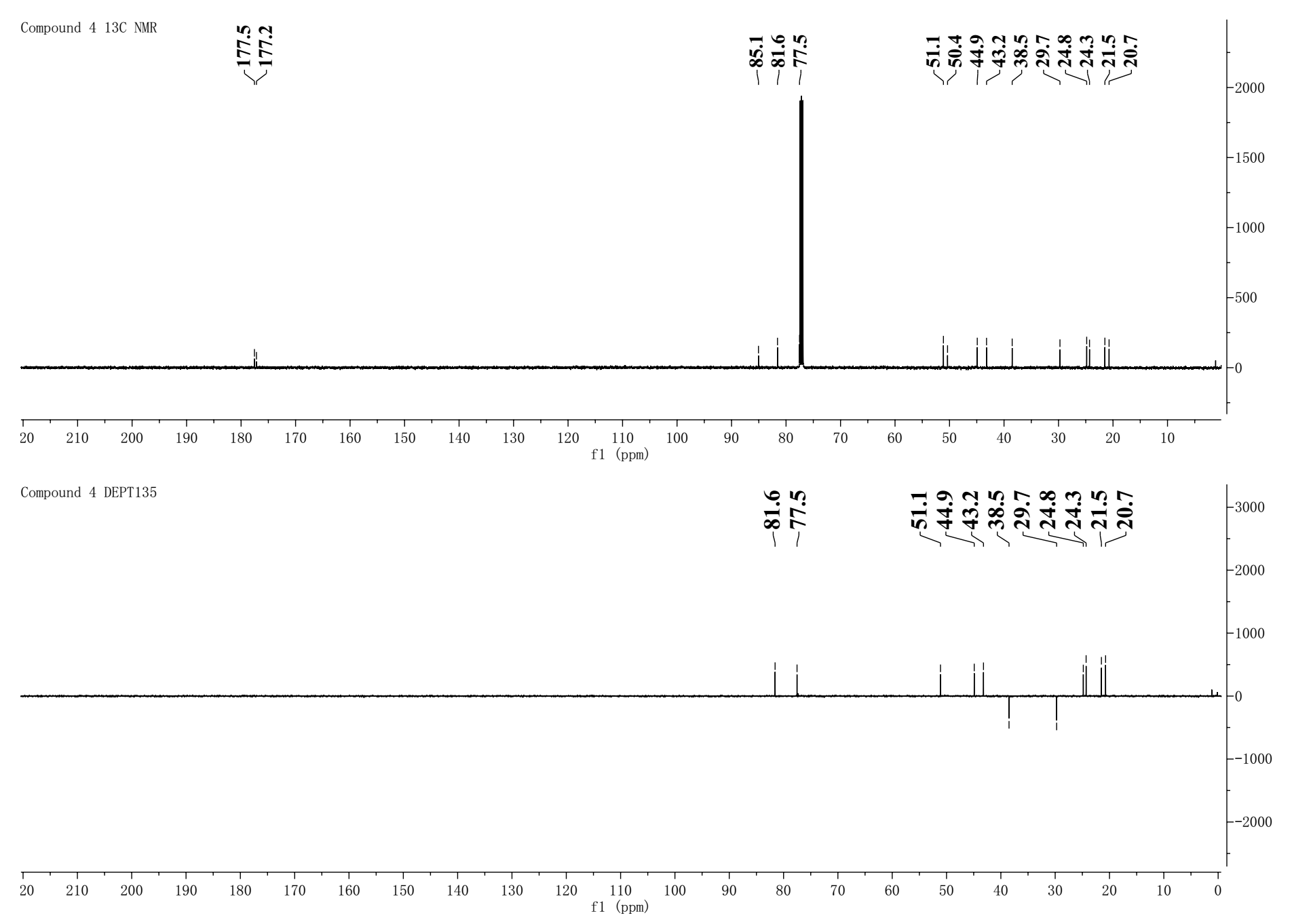


**Supplementary Figure 21.** The ^1^H-^1^H COSY (500 MHz, CDCl_3_-*d*) spectrum of **4**


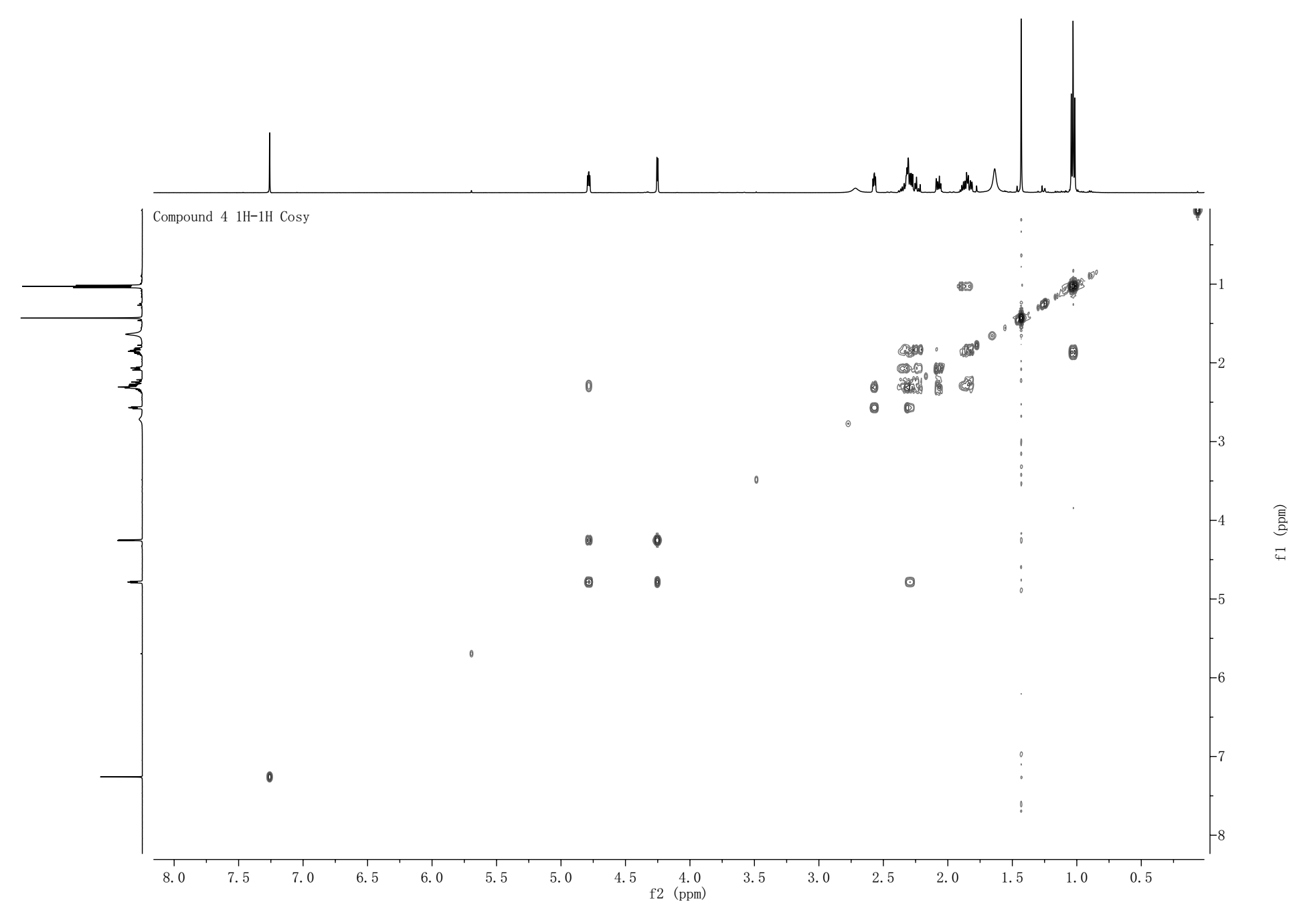


**Supplementary Figure 22.** The HSQC (500 MHz, CDCl_3_-*d*) spectrum of **4**


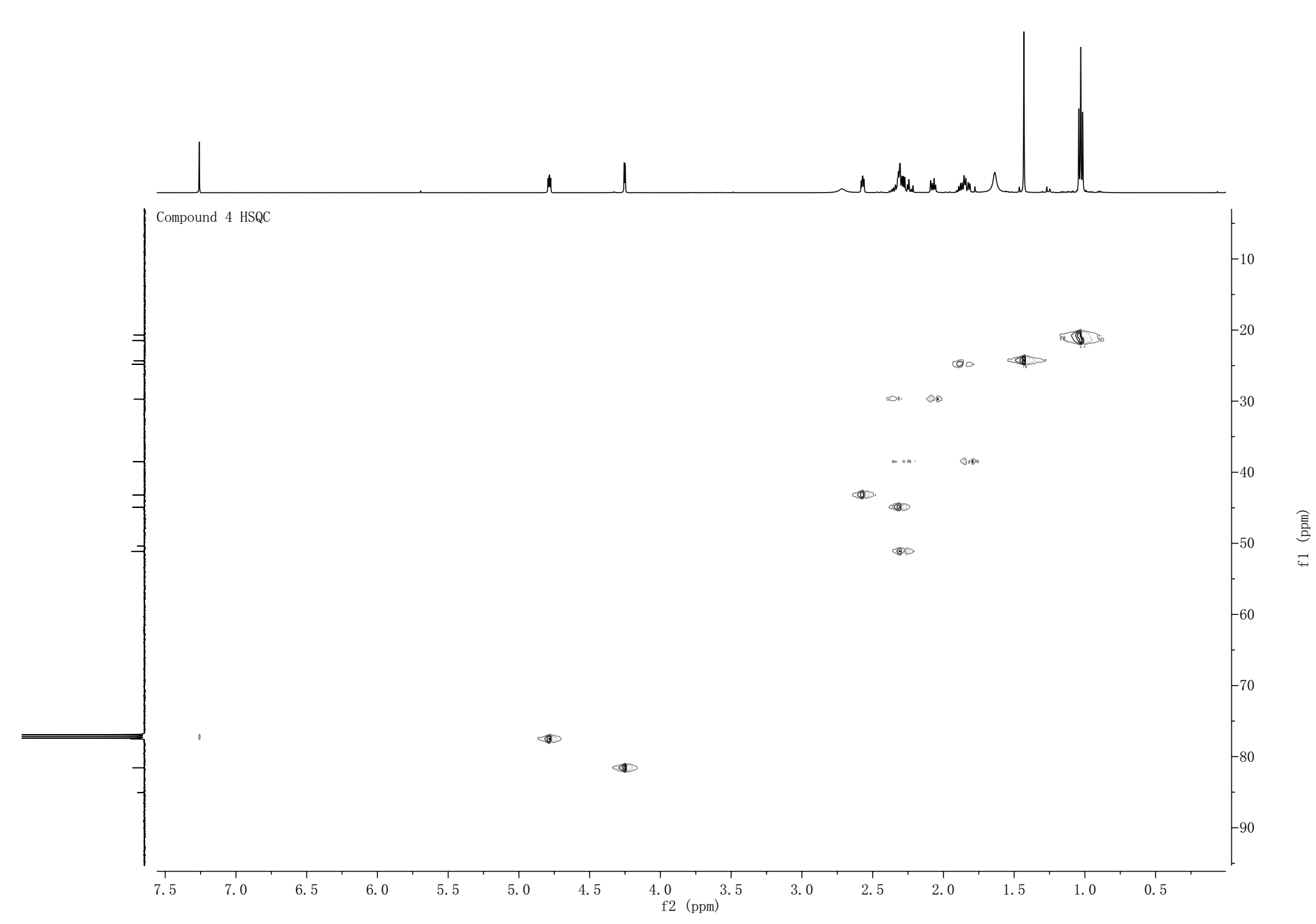


**Supplementary Figure 23.** The HMBC (500 MHz, CDCl_3_-*d*) spectrum of **4**


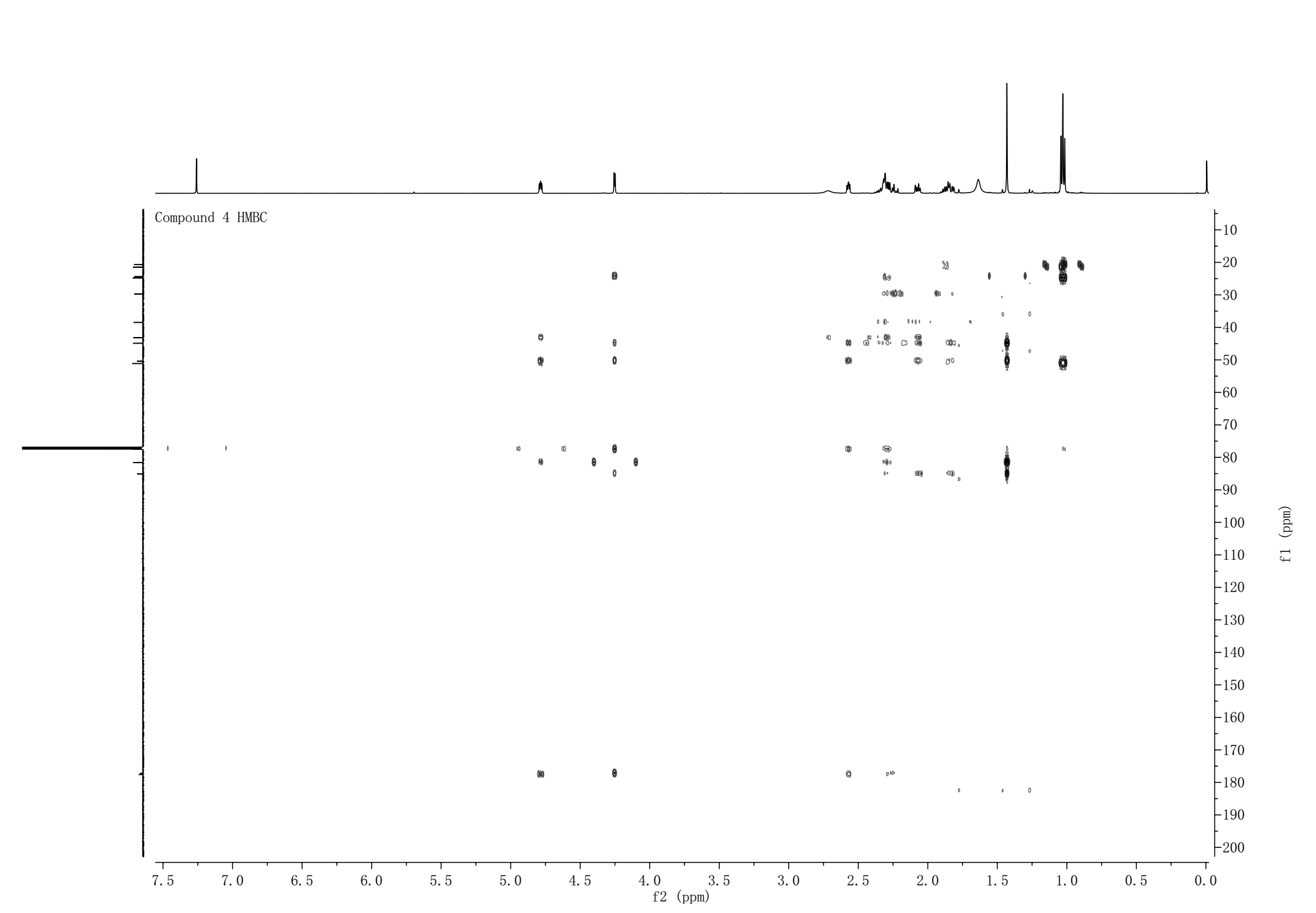


**Supplementary Figure 24**. Measured ECD curves of compounds **1** and **2**

**Supplementary Figure 25.** DFT-optimized structures for low-energy conformers of 1*R*, 2*S*, 3*R*, 4*S*, 5*R*, 6*S*, 9*S*-**3** at B3LYP/6-31G(d) level in methanol (PCM).

**Supplementary Table 1**. Cytotoxic andα-glycosidase inhibitory activities of **1**–**8**

| **Compound** | **IC_50_** values (*µ*M) | | | | | **IC_50_** values (mM) |
| --- | --- | --- | --- | --- | --- | --- |
|  | SGC-7901 | K562 | A549 | BEL-7402 | Hela | α-glycosidase |
| **1** | >50 | >50 | >50 | >50 | >50 | >1 |
| **2** | >50 | >50 | >50 | >50 | >50 | >1 |
| **3** | >50 | >50 | >50 | >50 | >50 | 0.97 |
| **4** | >50 | >50 | >50 | >50 | >50 | >1 |
| **5** | 17.30 | 10.39 | 29.03 | 20.13 | 22.19 | 0.03 |
| **6** | >50 | 28.23 | >50 | >50 | >50 | 0.68 |
| **7** | >50 | >50 | >50 | >50 | >50 | >1 |
| **8** | >50 | >50 | >50 | >50 | >50 | 0.30 |
| Cisplatin | 4.11 | 3.08 | 1.93 | 4.02 | 11.29 | ND^a^ |
| Acarbose | ND^a^ | ND^a^ | ND^a^ | ND^a^ | ND^a^ | 0.72 |

^a^ Not detected;
